# Effects of mirror-image nucleosides on DNA replication and transcription in human cells

**DOI:** 10.1016/j.jbc.2024.108139

**Published:** 2024-12-26

**Authors:** Zhaoyang Jin, Yifei Wang, Shuaishuai Cui, Yujian He, Li Wu

**Affiliations:** 1 School of Chemical Sciences, University of Chinese Academy of Sciences, Beijing, PR China; 2 College of Life Sciences, Hebei Normal University, Shijiazhuang, PR China; 3 State Key Laboratory of Natural and Biomimetic Drugs, School of Pharmaceutical Sciences, Peking University, Beijing, PR China; 4 School of Future Technology, University of Chinese Academy of Sciences, Beijing, PR China

**Keywords:** DNA replication, DNA transcription, human cells, mirror-image nucleosides, JAK–STAT signaling pathway

## Abstract

Mirror-image nucleosides, as potential antiviral drugs, can inhibit virus DNA polymerase to prevent virus replication. Conversely, they may be inserted into the DNA strands during DNA replication or transcription processes, leading to mutations that affect genome stability. Accumulation of significant mutation damage in cells may result in cell aging, apoptosis, and even uncontrolled cell division. We have previously explored the efficiency and fidelity of replication across mirror-image nucleosides within *Escherichia coli*, and this study focuses on human cells. We constructed several plasmid substrates, each carrying a specific mirror-image nucleoside, to investigate their impact on intracellular DNA replication and transcription processes. The results showed that in HepG2 cells, L-adenosine was the most potent substrate in inhibiting cell replication and transcription. L-cytidine exhibited the highest bypass efficiency in both template strands or nontemplate strands and had the most diverse mutation types. We also observed that L-cytidine induced immunoregulation of the JAK–STAT signaling pathway. Therefore, our results provide a theoretical basis for the disruptions caused by mirror-image nucleosides in replication and transcription and give us some understanding that mirror-image nucleoside drugs can cause cytotoxicity.

Natural nucleosides exist in the β-D configuration, while L-nucleosides exist in the opposite stereochemical configuration (Fig. S1). L-nucleosides and their derivatives are developed based on the structure of natural nucleosides and are widely studied for their potential applications in the treatment of cancer and viral diseases. In recent years, several nucleoside (nucleotide) analogs with selective inhibitory effects on viral replication have been discovered (1). These can be divided into two main categories: nucleosides with “non-natural” L-configuration and deoxyribonucleoside analogs with modified sugar configurations, represented by Telbivudine and Penciclovir, respectively. WHO estimates that approximately 296 million people worldwide are chronically infected with the hepatitis B virus (HBV) in 2019 (2), which can progress to chronic active hepatitis, cirrhosis, and hepatocellular carcinoma. Antiviral drugs are currently the main treatment of choice for long-term viral infections. However, chemotherapy for chronic HBV is a long-term process that increases the risk of viral resistance and cumulative drug toxicity.

Tibivudine (LdT) (3), a β-L-nucleoside analog, is a new drug used in hepatitis B. Tibivudine is phosphorylated by cellular kinases and converted to an active triphosphate form, which incorporation of viral DNA leads to termination of DNA strand synthesis, thereby inhibiting HBV replication. Besides, the antiviral drug lamivudine (β-L-nucleoside) can be inserted into DNA strands by mitochondrial DNA polymerase γ (pol γ). Limited mitochondrial DNA repair due to the inhibition of DNA polymerase γ (4) postingestion may induce cytotoxicity over an extended period, potentially resulting in long-term side effects. It has been shown that HIV-1 reverse transcriptase can bind 3 to 4 L-deoxythymidine monophosphate residues for continuous insertion into the DNA strand due to its low stereoisomerism. DNA polymerase α and *Escherichia coli* (*E. coli*) DNA polymerase I (Klenow fragment) can also bind at least two L-deoxythymidine monophosphate residues (5). L-nucleosides are administered to leukemic mice, and residual amounts are found in the liver of the diseased mice (6, 7). Prolonged ingestion of β-L-nucleoside analogs by patients may result in the introduction into the DNA strand, causing cytotoxic side effects and possibly even more serious diseases. Therefore, understanding the side effects or cytotoxicity of antiviral drugs produced intracellularly could help in screening for suitable antiviral drugs.

We investigated the intracellular repair of four structurally simple β-L-nucleosides (L-dA, L-dC, L-dT, and L-dG), which exhibit unusual specificity in inhibiting the replication of small DNA viruses, including HBV, Duck Hepatitis B virus, and Wood Chuck Hepatitis virus. These nucleosides have been identified as potent, selective, and highly specific inhibitors of HBV replication, demonstrating excellent antiviral potential in clinical trials (3). Surprisingly, L-nucleosides, incorporated into non-template strands can be repaired both *in vitro* and in *E. coli* (8). Given such phenomena, we plan to further investigate the bypass efficiency and fidelity of L-nucleosides during human cell replication and transcription. In this report, we systematically studied how the insertion of the four aforementioned lesions (L-dA, L-dC, L-dT, and L-dG) in the template strands (TSs) or non-template strands affects replication and transcription in HepG2 cells. We also explored the signaling pathways induced by L-cytidine during the repair in cells. Our current findings provide a theoretical basis for the long-term use of L-deoxyriboside analogs for toxic side effects arising from HBV or other viral infections.

## Result

### Construction of plasmids containing L-dA, L-dC, L-dT, and L-dG lesions

**Figure 1:**
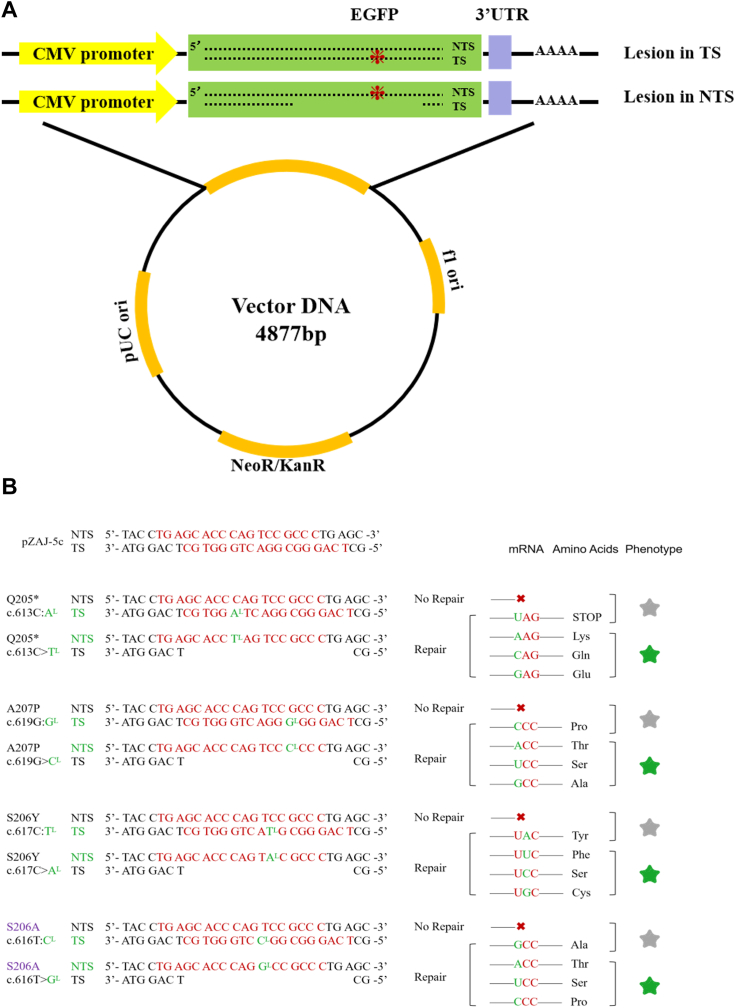
**Schematic representation of the location of lesion-containing constructs and experimental results.***A*, schematic diagram of plasmid construction. *B*, schematic representation of the fluorescent expression of the constructs. Strand break. *Green* fluorescence. *Purple* (S206A) indicates unsuitable for flow cytometry analysis.

This study aims to evaluate how different L-nucleoside lesions interfere with replication and transcription and to investigate the differences in transcription bypass efficiency between normal human cells and cancer cells. These lesions are located downstream of the cytomegalovirus promoter, which facilitates human RNA polymerase II (RNAPII) mediated transcription (Fig. 1*A*). To generate a reporter gene for direct detection of transcriptional mutagenesis (TM) and replication mutagenesis (RM) in human cells, we used a mutant form of EGFP encoding a truncated green fluorescent protein. Its DNA sequence allows for the specific insertion of oligonucleotides containing synthetic DNA lesions, due to the presence of adjacent Bpu10I restriction sites. This EGFP construct, when substituted at a specific site with any one of the other three nucleosides, results in a reconstructed EGFP fluorescence amino acid (Fig. 1*B*). Based on these characteristics, a method for direct detection of TM and RM occurring in human cells was established (9).

To detect fidelity in transcription, the method involves inserting lesions at the Nb.Bpu10I restriction site in the TS, and any misincorporation of nucleosides in the mRNA opposite the lesion will result in a reversal of the expected EGFP phenotype. Similarly, the detection of fidelity in replication can be achieved using this method by inserting the lesion at the Nt.Bpu10I restriction site in the NTS and removing the oligonucleotides (ODNs) from the corresponding TS to create a gapped plasmid (Fig. 1*B*). Full-length EGFP transcripts can only be produced after the missing TS fragment has been resynthesized, which requires lesion bypass. To prevent errors due to the host cells' self-replication, we used cells without the SV40T antigen, rendering the vector incapable of replicating within the cells.

**Figure 2:**
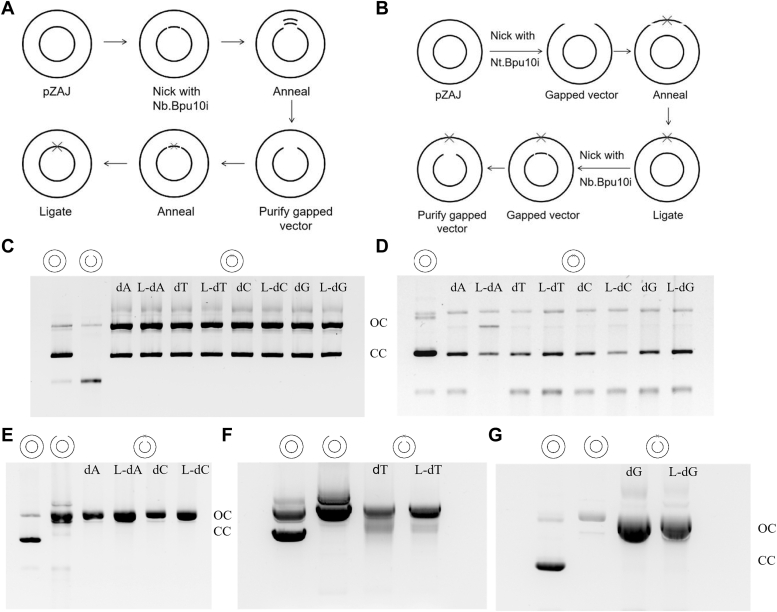
**Construction of plasmids containing mirror-image nucleosides and controls.***A*, steps for constructing mirror nucleosides into the TS of the plasmid. *B*, steps for constructing mirror nucleosides into the NTS of the plasmid. *C* and *D*, agarose gel analyses of insertion of the oligonucleotides into the TS of the correspondent gapped plasmid substrates. The former is the plasmid after synthesis by T4 DNA ligase and the latter after gel extraction and purification of the plasmid. *E*–*G*, agarose gel analysis of the gapped plasmids containing mirror-image nucleosides in the NTS. The pictogram above the gels indicates the topology of the main products, where mirror-image nucleosides are indicated by *crosses*. OC means open circular form, and CC means closed circular form. NTS, nontemplate strand; TS, template strand.

**Supporting Figure S2:**
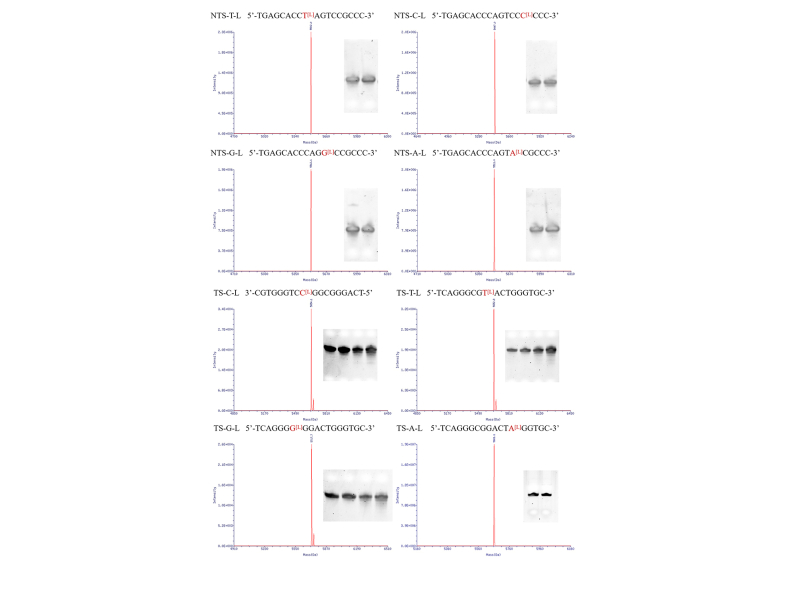


**Supporting Figure S3:**
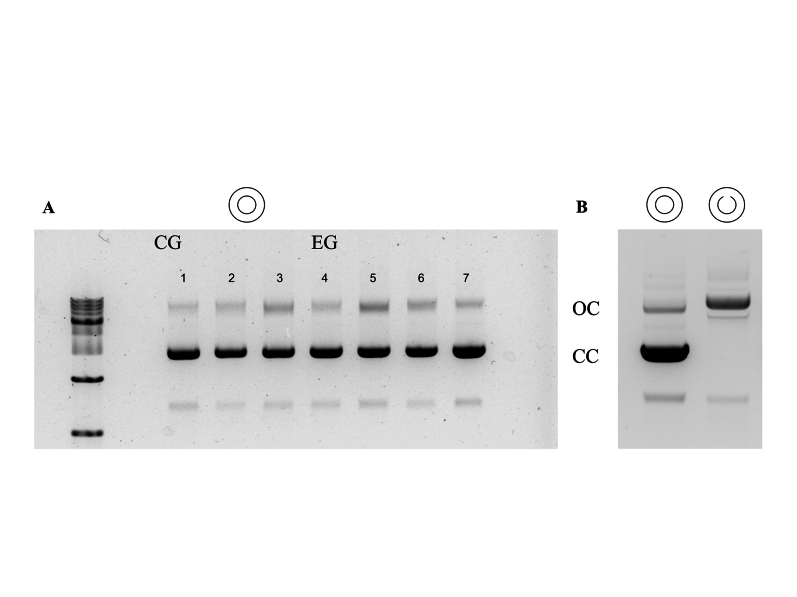


**Supporting Figure S4:**
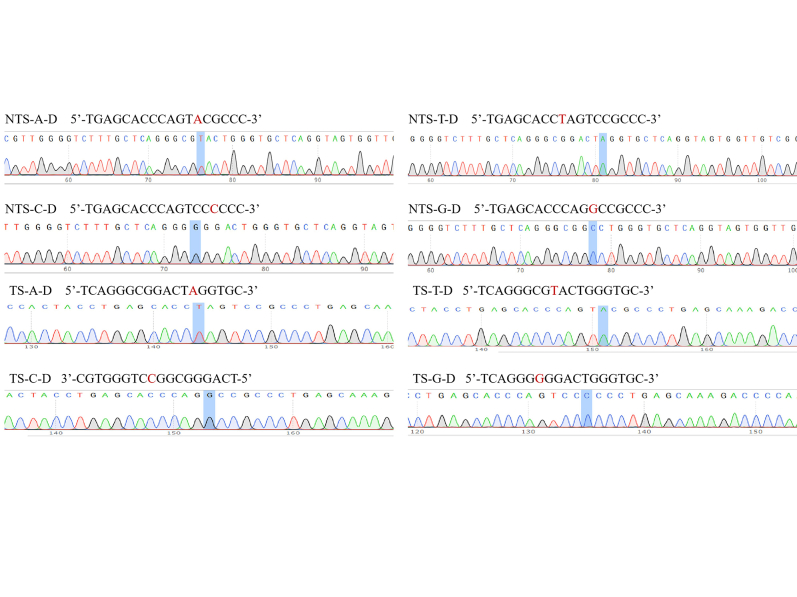


**Supporting Figure S5:**
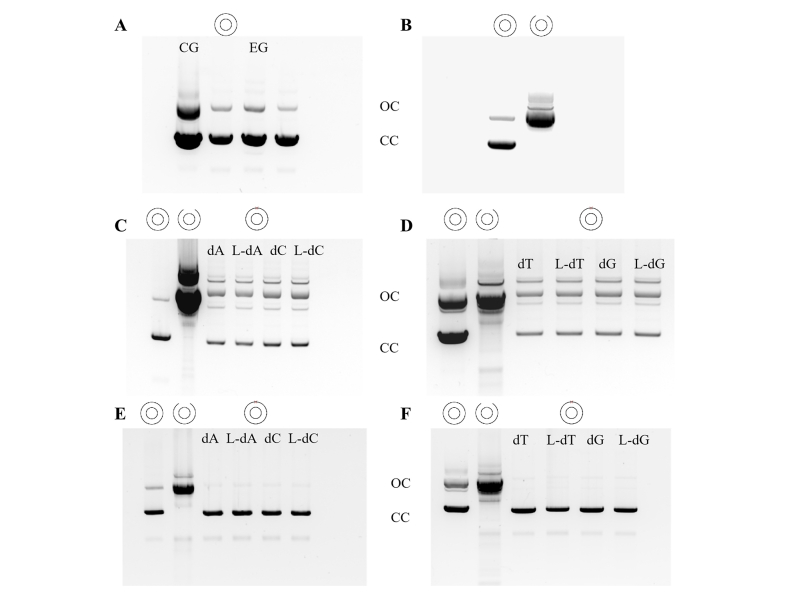


We used four reporter genes: EGFP Q205P, A207P, S206Y, and S206A (Fig. 1*B*) (9). Among these, EGFP Q205P, A207P, and S206Y mutants are considered suitable for detecting mutations in replication and transcription processes using flow cytometry, while the S206A mutant has limited capability. Therefore, we also chose an approach that utilizes targeted complementary DNA (cDNA) sequencing to obtain a more accurate distribution of mutation frequencies. First, we synthesized ODNs containing the lesion sites (L-nucleosides) (Table S1 and Fig. S2). The original plasmid pZAJ-5c was used as the initial construct reference (10, 11, 12). We constructed double-strand lesion-containing plasmids for transcriptional bypass detection, as well as corresponding control plasmids (Fig. 2*A*). Successfully transformed recombinant plasmids were obtained using gel extraction (Figs. 2, *C* and *D*, and S3). Since L-nucleosides cannot be validated by sequencing, the result of gel extraction was determined by sequencing the simultaneously performed undamaged recombinant plasmids (control group). The sequencing results confirmed that the plasmids were fully converted into the desired recombinant form without interference from other sequences (Fig. S4). We also constructed gapped plasmids for replication bypass detection, along with the corresponding control gapped plasmids (Fig. 2*B*). Successfully transformed closed circular plasmids were recovered using the same gel extraction method (Fig. S5, *A*–*F*). Using Bpu10I restriction endonuclease, gapped plasmids were generated (Fig. 2, *A*–*G*). Synthesis efficiency was determined by sequencing the control plasmid. The sequencing results confirmed that the plasmid had been completely transformed into the desired recombinant form without interference from other sequences (Fig. S4).

### Effect of L-dT, L-dA, L-dG, and L-dC on the bypass efficiency and fidelity of DNA replication in HepG2 cells

**Figure 3:**
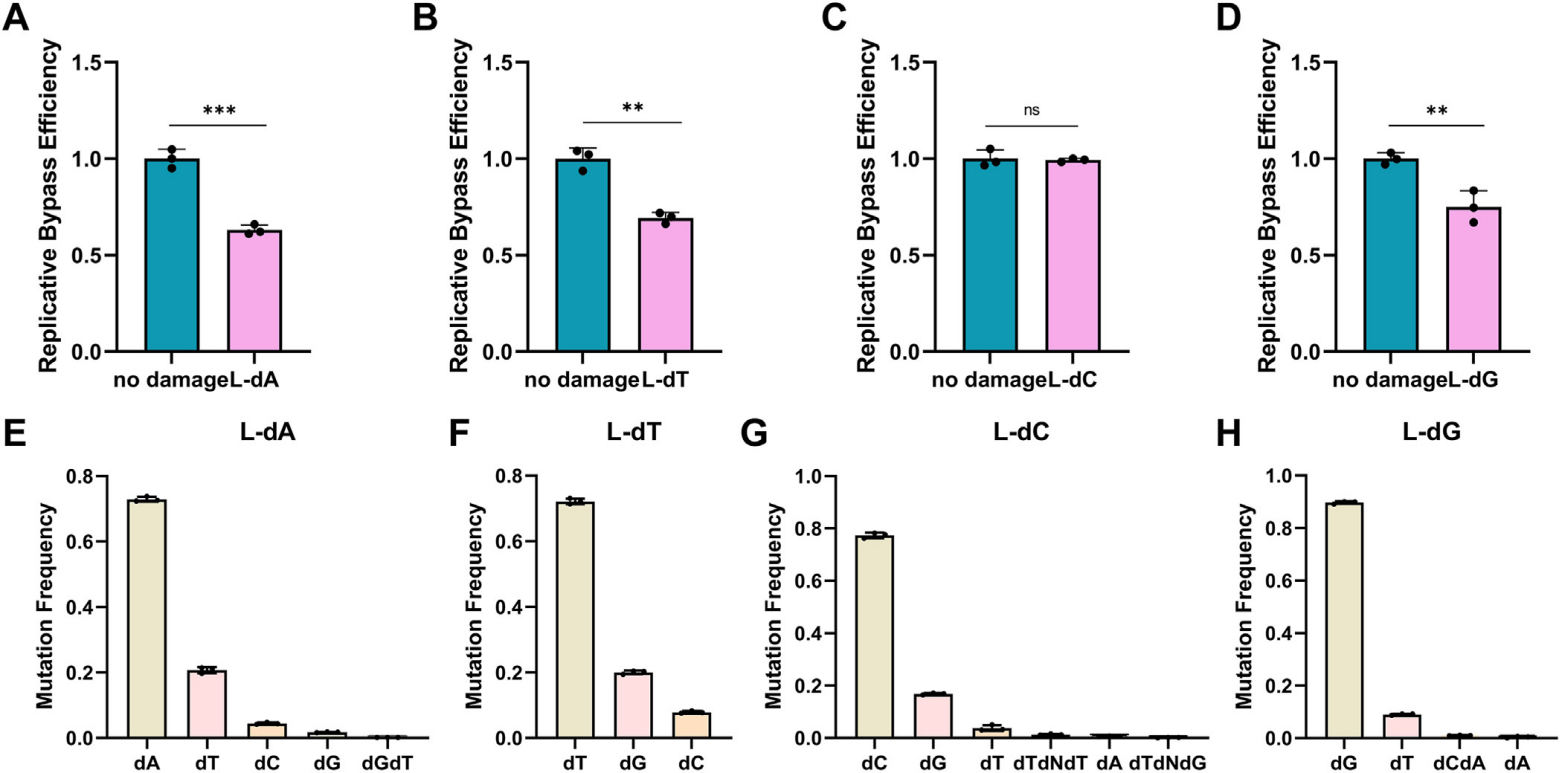
**Relative replication bypass efficiencies and mutation frequencies of mirror-image nucleoside in HepG2 cells.***A*–*D* are relative transcriptional bypass efficiencies. *E*–*H* are mutation frequencies.

The main purpose of this study is to evaluate how the introduction of four types of mirror-image nucleosides on the DNA strand interferes with the efficiency of DNA replication in HepG2 cells. Inserting mirror-image nucleosides into the NTS causes the TS to halt or undergo resynthesis at or near the lesion site. A full-length EGFP transcript is only produced once the missing fragments of the TS are resynthesized. If stalling occurs, it cannot be transcribed into mRNA. We separately transfected plasmids containing L-nucleosides at the special sites with plasmids containing pDsRed-monomer-N1 as an internal control gene into HepG2 cells at a fixed molar ratio. After transfection for 18 h, mRNA was extracted from HepG2 cells, and RT-PCR was used to selectively amplify the transcription products near the damaged strands. The degree of inhibition of DNA replication by these four mirror-image nucleosides was determined by comparison with the pDsRed-monomer-N1 plasmid internal reference gene. The blank control group indicates no influence by other genes. Subsequently, the experimental group and the control group were amplified using forward and reverse primers, and quantitative analysis was performed to calculate the relative bypass efficiency (RBE). The RBE was calculated using the following formula, RBE = (lesion signal/reference signal)/(unmodified control signal/reference signal). Our results showed that, except for L-dC, all three types of mirror-image nucleosides significantly blocked DNA replication in HepG2 cells. In HepG2 cells, the bypass efficiencies of L-dA, L-dT, L-dC, and L-dG were 63.14%, 69.37%, 99.27%, and 75.09%, respectively (Fig. 3, *A*–*D*). L-dA had the highest inhibition rate on DNA replication, while L-dC had the lowest inhibition rate in the cells. Overall, the bypass efficiency of HepG2 cells is generally higher, which is consistent with previous observations in *E. coli* experiments (8, 13).

**Figure 4:**
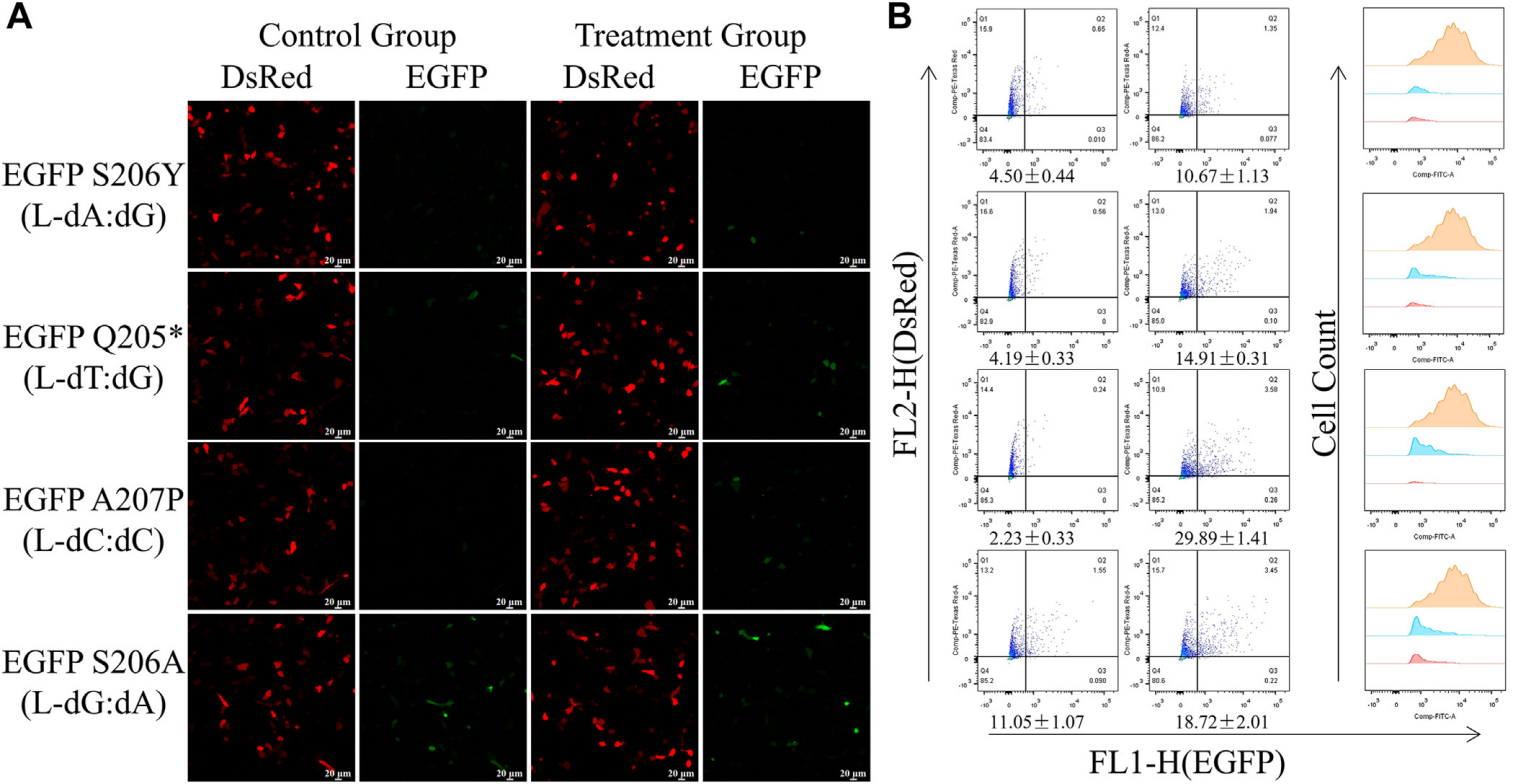
**Detection of mirror-image nucleosides in reporter genes by restoration of fluorescent phenotypes for replication mutagenesis.***A*, replication mutagenesis (RM) was visualized by confocal microscopy as a reversion of the nonfluorescent EGFP phenotype. HepG2 cells were transfected with the indicated EGFP constructs, and DsRed-monomer was present as a transfection marker. *B*, transfection-based reporter assay for direct detection of RM templated by synthetic DNA lesions incorporated into vector DNA: repair of the 18-nt gap by resynthesis of the missing TS fragment in a construct encoding functional EGFP with EGFP Q205∗ without modification (c.6T) or with mirror-image nucleosides (c.6L-dT) at nucleoside 613; EGFP A207P without modification (c.6C) or with mirror-image nucleosides (c.6L-dC) at nucleoside 619; EGFP S206Y without modification (c.6A) or with a mirror-image nucleoside (c.6L-dA) at nucleoside 617; and EGFP S206A without modification (c.6G) or with a mirror-image nucleoside (c.6L-dG) at nucleoside 616 were used for detection of mutagenic RM on four templates. HepG2 cells were transfected with the constructs shown and DsRed-monomer expression vectors as transfection markers. Mutation frequency was calculated as the ratio (mean ± SD) of EGFP-positive cells (*upper right quadrant*, UR) to total transfected cell counts (UR + UL) in n = 3 independent experiments. Scatter plots were gated by DsRed fluorescence to generate EGFP fluorescence distribution maps and calculate median FL1-h. The grouped plots show data from representative experiments. template strand.

**Supporting Figure S6:**
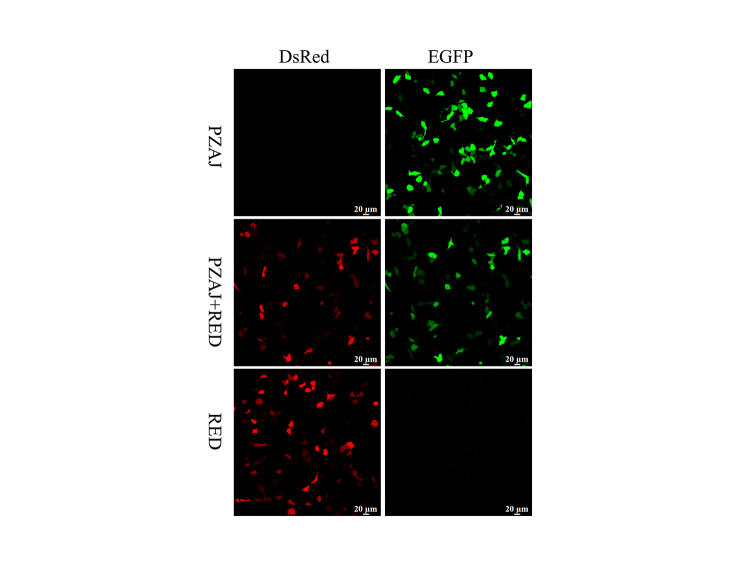


**Supporting Figure S7:**
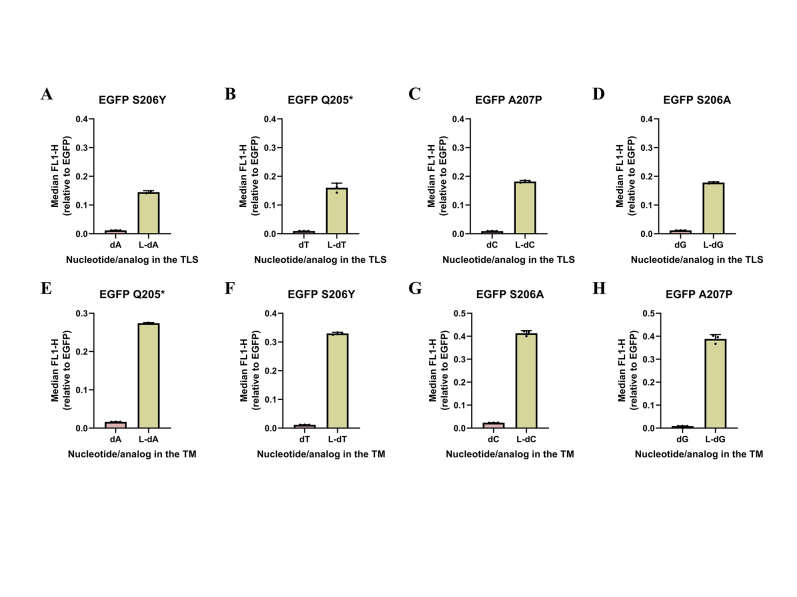


The next step is to use targeted gene sequencing and flow cytometry analysis to assess the mutagenicity of the four mirror-image nucleosides. Data from targeted gene sequencing shows that in HepG2 cells, L-dC exhibits high mutagenic potential and is prone to strong misincorporation (Fig. 3*G*). It generates a frequency of 16.80% for L-dC→dG substitution at the site of the lesion. Additionally, it also causes substitutions such as L-dC→dT, L-dC→dTdNdT, L-dC→dA, and L-dC→dTdNdG (Fig. 3*G*). L-dT has a moderate mutagenic potential with a mutation rate of 27.87% (Fig. 3*F*). L-dA has a moderate mutagenic potential (27.10%), with L-dA→dT substitution at 20.76%, L-dA→dC substitution at 4.44%, L-dA→dG substitution at 1.74%, and L-dA→dGdT tandem substitution at 0.15% (Fig. 3*E*) (14). L-dG caused mostly L-dG→dT substitutions (8.98%), while L-dG→dCdA and L-dG→dA substitutions were less frequent (1.00% and 0.36%, respectively). (Fig. 3*H*). These findings are inconsistent with previous studies in *E. coli*, possibly due to different mechanisms in human cells compared to prokaryotic cells (15). In our mutation analysis, we observed that damage at a single site may lead to mutations in adjacent nucleosides, involving up to three nucleosides, as seen in the case of L-dC. Additionally, some large-scale nucleoside deletions were observed during the replication bypass process, with the number of deletions ranging from 31 to 43 bp, despite the extremely low deletion proportion (Table S2). The reversal of the fluorescence phenotype of EGFP was subsequently detected using flow cytometry. Targeted gene sequencing provides a “relative mutation frequency” for replication bypass, while flow cytometry analysis compares repaired DNA with a WT EGFP construct, taking into account blockages occurring during DNA replication. Multiplying the mutation frequency by the bypass efficiency yields the percentage of mutation-restricted fragments in the damaged genome. Based on the expression of normal EGFP plasmids in flow cytometry transfection, EGFP signals were detected in approximately 86% of transfected cells. Except for the construct containing L-dG (S206A), the proportion of EGFP-positive cells for the other three constructs was negligible. Compared to EGFP A207P c.619C (Figs. 4, S6 and S7), the EGFP A207P c.619L-dC construct showed a significantly increased proportion of cells with reversed fluorescence phenotype, at approximately 29.89% ± 1.41%. The remaining two constructs, EGFP S206Y c.617L-dA (10.67% ± 1.13%) and EGFP Q205∗ c.613L-dT (14.91% ± 0.31%) (Figs. 4, S6 and S7), were consistent with the sequencing results (Fig. 3, *E*–*H*). In conclusion, the results show that L-dC has extremely strong bypass efficiency during replication, while also exhibiting a high mutation frequency.

### Effect of L-dT, L-dA, L-dG, and L-dC on DNA transcription bypass efficiency and fidelity in HepG2 cells

**Figure 5:**
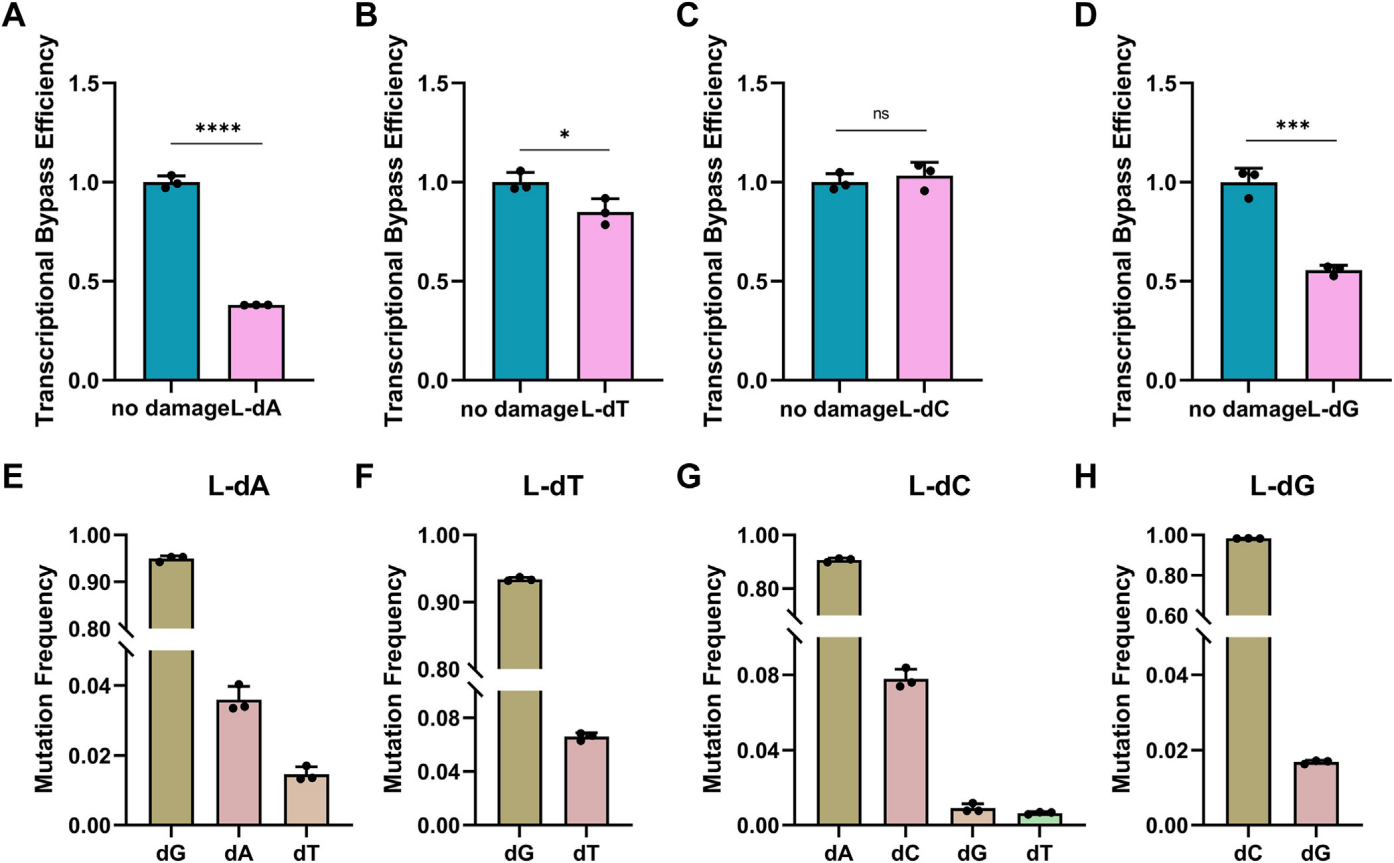
**Relative transcriptional bypass efficiencies and mutation frequencies of mirror-image nucleoside in HepG2 cells.** *A*–*D* are relative transcriptional bypass efficiencies. *E*–*H* are mutation frequencies.

**Supporting Figure S8:**
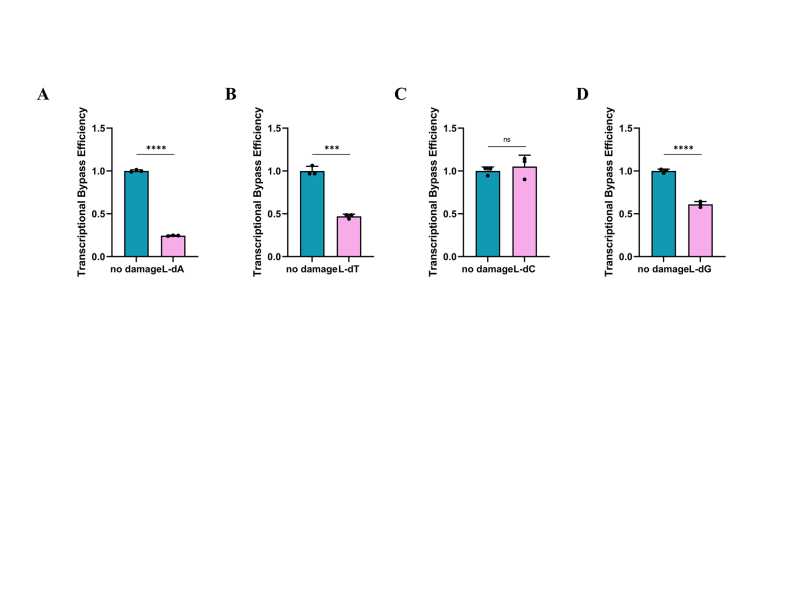


The main objective of this study is to evaluate how the introduction of four different mirror-image nucleosides on the DNA strands interfere with DNA transcription bypass efficiency and fidelity in HepG2 cells. We have made a bold attempt to explore the plasmid construction of TM, using the pZAJ-5c plasmid with EGFP Q205∗ c613C:L-dA, EGFP A207P c619G:L-dG, EGFP S206Y c617C:L-dT, and EGFP S206A c616T:L-dC to generate mismatched base complementation, investigating whether the bypass efficiency and fidelity of nucleosides are related to recognition of mirror-image nucleosides or base pairs. These plasmids, along with corresponding plasmids containing L-nucleosides at specific sites, were then co-transfected into HepG2 and L02 cells at a fixed molar ratio, with DsRed-Monomer plasmid serving as an internal control gene. Subsequently, experimental and control groups were amplified using forward and reverse primers, followed by quantitative analysis to calculate the RBE. Our research results indicate that all four mirror-image nucleosides to some extent impede DNA transcription in both L02 and HepG2 cells. In L02 cells, the bypass efficiencies of L-dA, L-dT, L-dC, and L-dG are 24.44%, 47.05%, 105.23%, and 61.01%, respectively (Fig. S8). In HepG2 cells, the bypass efficiencies of L-dA, L-dT, L-dC, and L-dG are 38.01%, 84.97%, 103.29%, and 55.50%, respectively (Fig. 5, *A*–*D*). In L02 cells, L-dA exhibits the highest inhibition of DNA transcription, while L-dC shows the lowest inhibition in the cells. In HepG2 cells, consistent with L02 cells, L-dA demonstrates the highest inhibition of DNA transcription, while L-dC exhibits the lowest inhibition in the cells. Moreover, the bypass efficiency of normal and cancer cells was similar, indicating that some repair mechanisms are common in cells.

**Figure 6:**
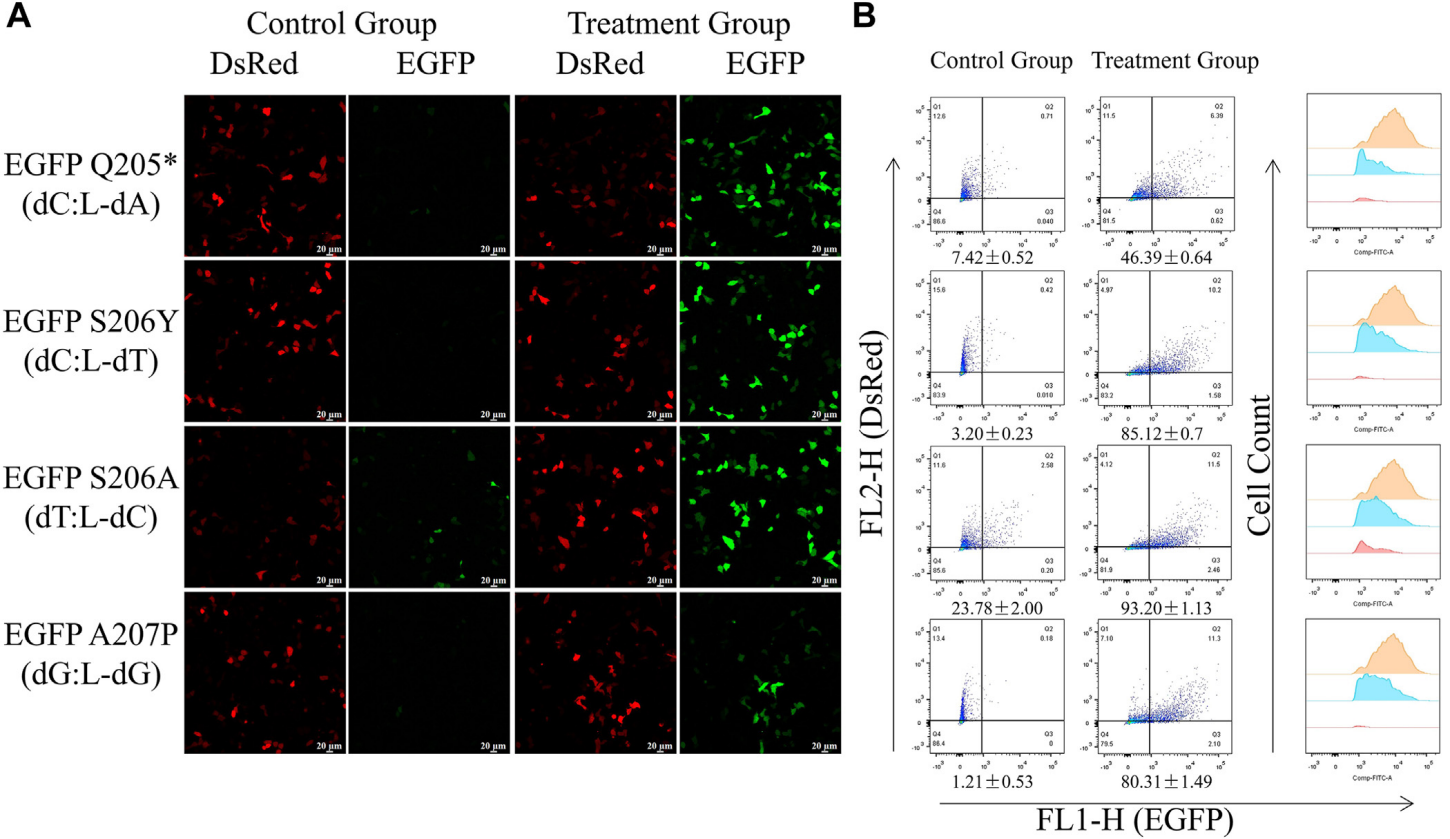
**Detection of mirror-image nucleosides in reporter genes by restoration of fluorescent phenotypes for mutagenic transcriptional mutagenesis.***A*, transcriptional mutagenesis (TM) was visualized by confocal microscopy as a reversion of the nonfluorescent EGFP phenotype. HepG2 cells were transfected with the indicated EGFP constructs, and DsRed-monomer was present as a transfection marker. *B*, transfection-based reporter assay for direct detection of transcriptional mutagenesis (TM) templated by synthetic DNA lesions incorporated into vector DNA: with EGFP Q205∗ without modification (c.6T:A) or with mirror-image nucleosides (c.6C:L-dA) at nucleoside 613; EGFP A207P without modification (c.6C:G) or with mirror-image nucleosides (c.6G:L-dG) at nucleoside 619; EGFP S206Y without modification (c.6A:T) or with a mirror-image nucleoside (c.6C:L-dT) at nucleoside 617; and EGFP S206A without modification (c.6G:C) or with a mirror-image nucleoside (c.6T:L-dC) at nucleoside 616 were used for detection of transcriptional mutagenesis (TM) on four templates. HepG2 cells were transfected with the constructs shown and DsRed-monomer expression vectors as transfection markers. Mutation frequency was calculated as the ratio (mean ± SD) of EGFP-positive cells (*upper right quadrant*, UR) to total transfected cell counts (UR + UL) in n = 3 independent experiments. Scatter plots were gated by DsRed fluorescence to generate EGFP fluorescence distribution maps and calculate median FL1-h. The grouped plots show data from representative experiments.

We used targeted gene sequencing and flow cytometry analysis to assess the fidelity of four mirror-image nucleosides. Data from targeted gene sequencing revealed a high proportion of mutations for all four mirror-image nucleosides in HepG2 cells. Interestingly, the primary mutation types observed in the four mirror-image nucleosides were the replacement of complementary bases at their respective positions. The replacement rates were highest for L-dA→dG (94.95%) (Fig. 5*E*), L-dT→dG (93.40%) (Fig. 5*F*), L-dC→dA (90.68%) (Fig. 5*G*), and L-dG→dC (98.32%) (Fig. 5*H*). Incorrect nucleosides are frequently associated with complementary bases during the transcription process. Among the four mirror-image nucleosides, L-dC showed the highest frequency of mutation types, with a replacement rate of 0.90% for L-dC→dG. Except for L-dT→dT (6.60%), the other two mirror-image nucleosides also showed dT replacements, with replacement rates of 1.46% (L-dA→dT) and 0.64% (L-dC→dT) (Fig. 5, *E*–*G*). In addition, some large-scale nucleoside deletions were observed during TM, with deletions ranging from 36 to 43 bp (Table S2). Similar to the principles of RM flow cytometry, the results of flow cytometry include the frequency of blockages occurring during resynthesis. Therefore, multiplying the obtained mutation frequency by the bypass efficiency yields the percentage of mutation-restricted fragments in the total amount of damaged genomic DNA. Based on the expression of the normal EGFP plasmid after flow cytometry transfection, EGFP signals were detected in approximately 78% of transfected cells. Except for the EGFP S206A c.616T:L-dC construct, which was not suitable for flow cytometry analysis, the proportion of EGFP-positive cells for the other three constructs was negligible (9). Compared to EGFP S206Y c.617A:T, EGFP Q205∗ c.613T:A, and EGFP A207P c.619C:G, the EGFP S206Y c.617C:L-dT, EGFP Q205∗ c.613C:L-dA, and EGFP A207P c.619G:L-dG constructs showed a significant increase in the proportion of cells displaying a reversed fluorescent phenotype, with approximately 85.12% ± 0.7%, 46.39% ± 0.64%, and 80.31% ± 1.49%, respectively (Fig. 6, *A* and *B*, S6 and S7). These results were generally consistent with the sequencing results except for slight differences in L-dG (Fig. 5, *E*–*H*). In summary, based on the sequencing results, L-dC exhibits a remarkably strong bypass efficiency in the TS. For the repair of mirror-image nucleosides in the transcribed strands, the effect of the complementary base is greatest. Therefore, we speculate that mirror-image nucleotides can be excised through the nucleotide excision repair pathway or other mechanisms, with the gap resynthesized using the complementary strands as a template.

### Screening of differentially expressed genes after transcriptome sequencing and their functional analysis

**Supporting Figure S9:**
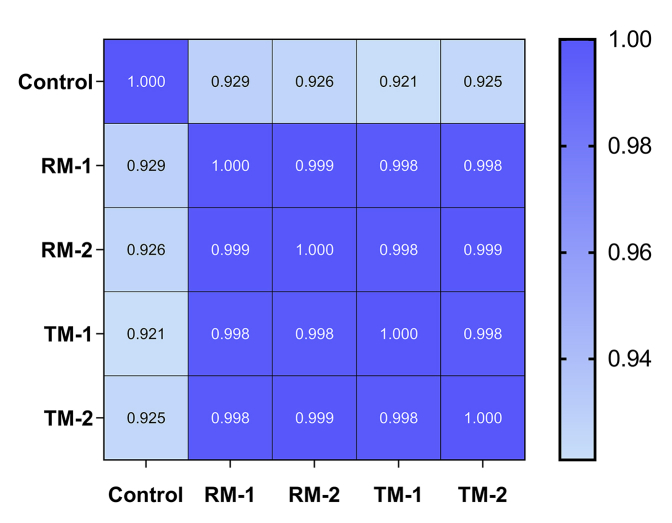


**Supporting Figure S10:**
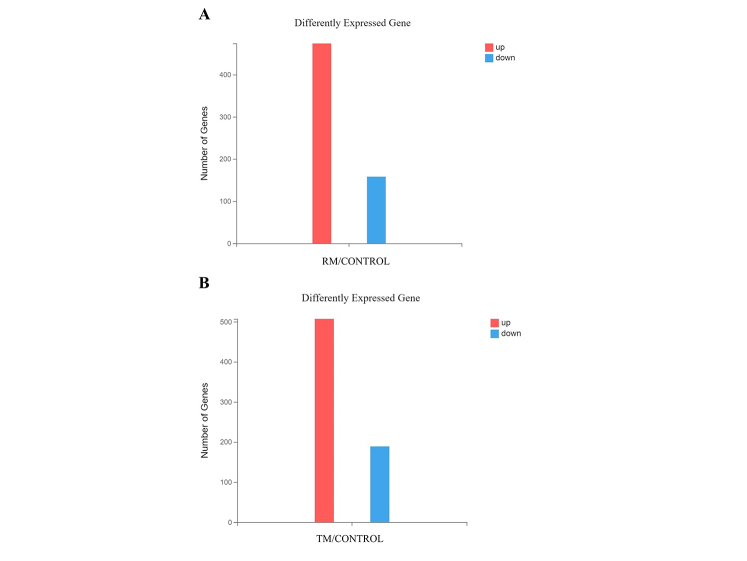


To investigate this phenomenon, we transfected cells with plasmids containing L-dC, which have the highest bypass efficiency in both TS and NTS. Cells transfected with normal plasmids were used as the control group. The average alignment rate of the samples to the genome reaches 93%, using Cutadapt to remove sequences with adapter contamination at the 3′ end, eliminating sequences with an average quality score lower than Q20. Through the merging and assembly of transcriptome data (Table S3), a total of 16,882 genes were detected. Statistical analysis of the genes in all cell samples was performed. The results show a high degree of similarity between samples of the same type (Fig. S9), which fulfills the requirements for subsequent studies. Gene expression differences were analyzed between experimental and normal cells with L-dC inserted into the TS and NTS (Fig. S10). Differential expression genes were selected based on fold change (FC) and *p* value criteria: the criteria for selection were |log2(FC)| > 1 for FC and a significance level of *p* < 0.05. There are 696 differentially expressed genes between cells with L-dC insertions in the TS and normal cells, with 507 (72.84%) upregulated genes and 189 (27.16%) downregulated genes (Fig. S10*B*). Similarly, there are 632 differentially expressed genes between the cells with L-dC insertions in the NTS and normal cells, with 474 (75.00%) upregulated genes and 158 (25.00%) downregulated genes (Fig. S10*A*).

**Figure 7:**
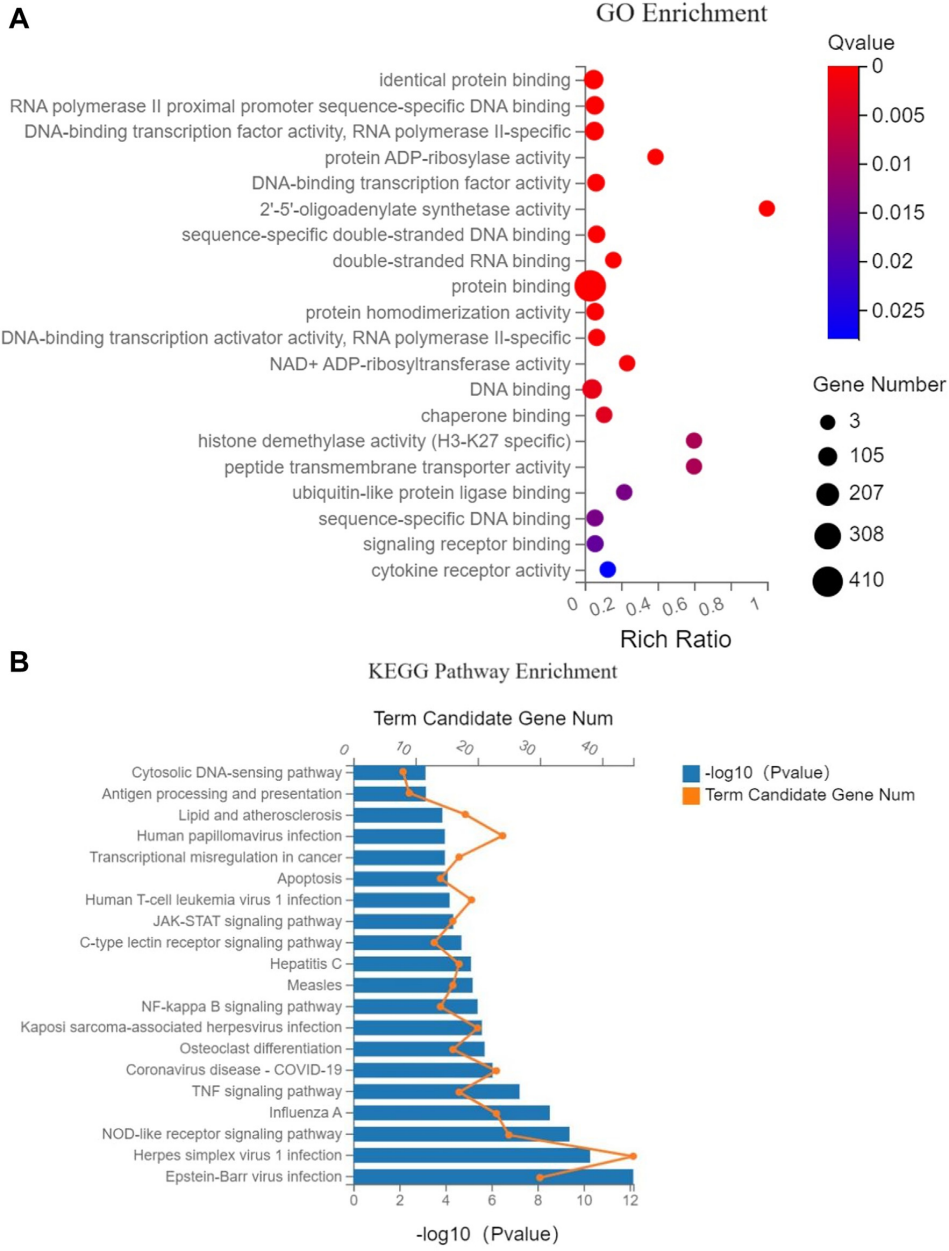
**Plot of GO and KEGG enrichment results for upregulated expressed genes after TM *versus* control.***A* is GO enrichment and *B* is KEGG enrichment.

**Supporting Figure S11:**
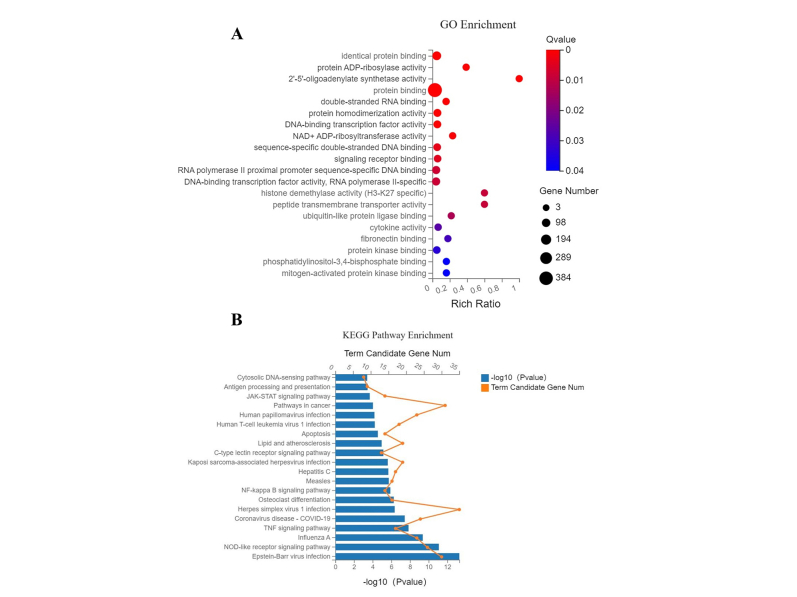


To investigate the differential gene expression between cells with L-dC insertions in the TS and control cells, Gene Ontology (GO) enrichment analysis was performed on the upregulated genes. A total of 693 GO terms were enriched, and the top 20 GO terms with the smallest Q values were selected for visualization (Fig. 7*A*). The *y*-axis represents the GO terms, while the *x*-axis and bubble size represent the number of genes belonging to the GO term in the target gene set. The color of the bubbles indicates the significance of enrichment, that is, the magnitude of the Q value. According to the GO database, gene sets enriched in terms such as RNA polymerase II proximal promoter sequence-specific DNA binding (containing 68 genes), DNA-binding transcription factor activity, RNA polymerase II–specific (containing 69 genes), DNA-binding transcription factor activity (containing 43 genes), sequence-specific dsDNA binding (containing 38 genes), dsRNA binding (containing 12 genes), DNA-binding transcription factor activity, RNA polymerase II–specific (containing 31 genes), and DNA binding (containing 94 genes) ranked high. Similarly, to understand the differential gene expression between cells with L-dC insertions in the NTS and control cells, GO enrichment analysis was conducted on the upregulated genes. A total of 670 GO terms were enriched, and the top 20 GO terms with the smallest Q values were visualized (Fig. S11*A*). Among these, gene sets enriched in terms such as dsRNA binding (containing 12 genes), DNA-binding transcription factor activity (containing 39 genes), sequence-specific dsDNA binding (containing 32 genes), RNA polymerase II proximal promoter sequence-specific DNA binding (containing 53 genes), and DNA-binding transcription factor activity, RNA polymerase II–specific (containing 55 genes) ranked high.

Kyoto Encyclopedia of Genes and Genomes (KEGG) pathway enrichment analyses yielded a total of 260 and 269 enriched pathways, corresponding to the results of the experimental group of cells for the detection of TM and RM compared to the control group, respectively (Figs. 7*B* and S11*B*). The *y*-axis represents the pathway names, while the *x*-axis and the size of the bars represent the number of genes belonging to the pathway in the target gene set. The length of the bars indicates the significance of enrichment, that is, the magnitude of the *p* value. Major pathways associated with both TM and RM experiments include the JAK–STAT signaling pathway, NF-kappaB signaling pathway, and TNF signaling pathway, suggesting that the repair process of mirror-image nucleosides may achieve its purpose through interactions with these signaling pathways.

**Supporting Figure S12:**
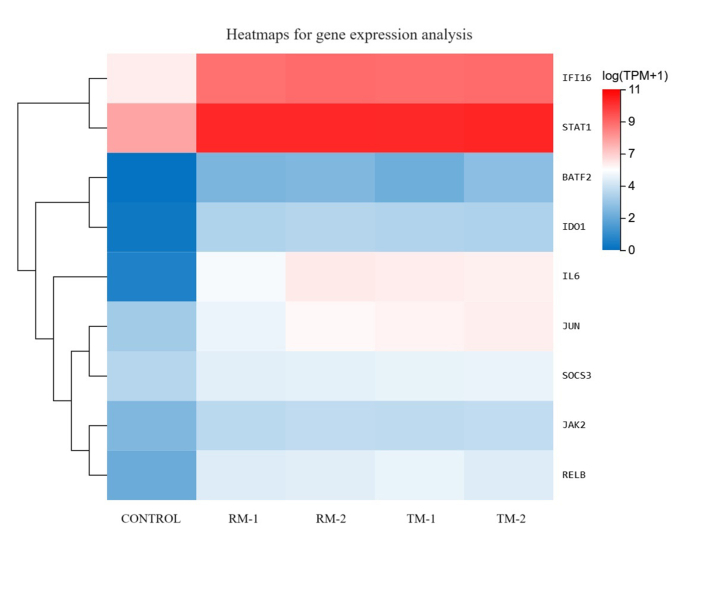


By comparing the transcriptome data of the experimental group of cells for the detection of TM and RM compared to the control group, 18 upregulated genes potentially associated with mirror-image nucleoside repair were identified (Table S4). Among these, four genes (gene3569, gene3717, gene9021, and gene6772) were found to be highly expressed in both experimental groups through KEGG pathway enrichment analysis, and they all play crucial roles at key points in the JAK–STAT signaling pathway. Furthermore, four genes (gene116071, gene3428, gene5971, and gene3725) related to RNA polymerase II repair were identified from the GO enrichment analysis of RNA polymerase II proximal promoter sequence-specific DNA binding. A cluster heat map analysis was performed on these eight genes, revealing a high similarity in gene expression levels between the experimental groups TM and RM (Fig. S12), suggesting a similar impact of L-dC–induced repair *in vivo*.

**Supporting Figure S13:**
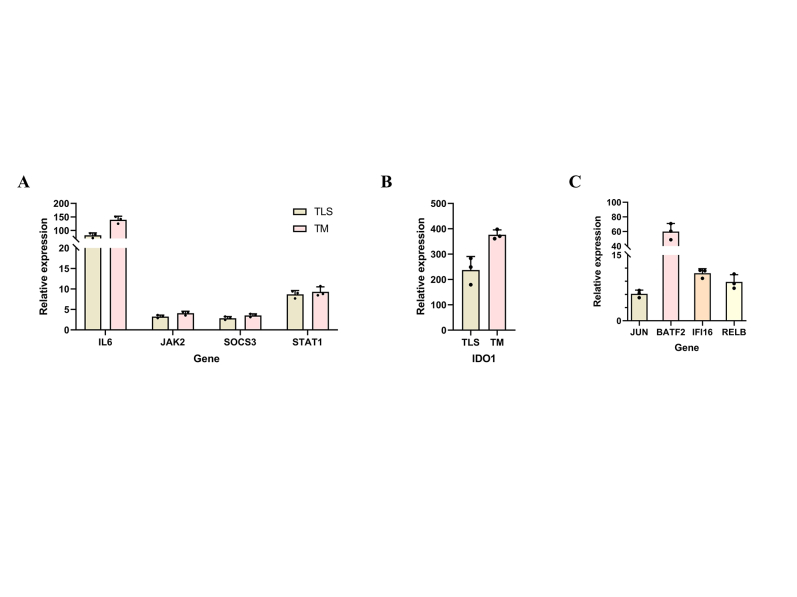


To further validate the accuracy of the differentially expressed genes selected above, reverse transcription-quantitative PCR analysis was conducted (Fig. S13). Four differentially expressed genes related to both RM and TM showed a significant upregulation, with substantial FCs. Among these, interleukin 6 (IL6) was upregulated 81.91-fold in RM and 139.17-fold in TM, JAK2 was upregulated 3.25-fold in RM and 4.08-fold in TM, SOCS3 was upregulated 2.85-fold in RM and 3.54-fold in TM, and STAT1 was upregulated 8.70-fold in RM and 9.31-fold in TM, indicating the reliability of the transcriptome sequencing data. It has been found that repair between transcribed strands is closely related to RNA polymerase II, and the TM experimental group ranked higher than the RM experimental group in the GO enrichment plot for the entry (RNA polymerase II proximal promoter sequence-specific DNA binding). Therefore, qPCR validation was performed on the upregulated genes related to LC-induced repair on the TS strand, where JUN was upregulated 6.15-fold, BATF2 was upregulated 59.99-fold, IFI16 was upregulated 10.85-fold, and RELB was upregulated 8.85-fold. We also found that among the genetically upregulated differential genes, the indoleamine 2,3-dioxygenase 1 gene had higher folds of upregulation, 376.47-fold in TM, and 237.28-fold in RM.

## Discussion

The basic functions of DNA in all cell types involve replication and transcription. Extensive experimental evidence suggests that DNA damage occurring within gene replication or transcription units often affects these functions, in which translesion synthesis (TLS) DNA polymerases (14) and RNA polymerase II (16, 17, 18) are involved in the repair of lesions to the mirror-image nucleoside. This scenario poses a potential concern for gene regulation and spatial regulation required for effective gene expression. In this study, we examined the bypass efficiency and fidelity of four mirror-image nucleosides with different spatial site resistance during the processes of replication and transcription in human cells.

In the study of NTS doped with mirror-image nucleosides, previous research (13) has found that L-dA has the lowest bypass efficiencies in *E. coli* (74%), while L-dT has the highest bypass efficiencies (99%), and only these two nucleosides exhibit mutations. However, *in vitro* studies using B-family polymerase *Vent (exo-)* DNA polymerase has shown that both L-dA and L-dG can induce mutations, but their bypass efficiencies are the lowest (8). In HepG2 cells, it was found that the low bypass efficiencies of L-dA (Fig. 3*A*) are consistent with previous research findings in *E. coli* and *in vitro*. The Y-family DNA polymerases possess larger active sites than replicative polymerases, allowing them to accommodate distorted DNA structures (19, 20). They feature stubbier finger and thumb domains, reduced contact with DNA and incoming nucleotides, and exhibit lower fidelity. This property enables them to polymerize on lesion. Furthermore, Y-family polymerases are involved in TLS by inserting nucleotides opposite the damaged template (19). We speculate that Y-family DNA polymerases (21), such as Pol η, Polι, and Polκ, assist in repairing mirrored nucleotides with varying effectiveness. They are less effective at repairing L-dA lesion but more effective for L-dC. In addition to restoring to the corresponding normal base, L-dT can also mutate into dG and dC (Fig. 3*F*). This is consistent with the induced mutagenesis results of O^2^-POB-dT in *E. coli* (22, 23). In addition, mirror-image nucleosides usually have the highest chance of being correctly repaired, suggesting that they can be accurately recognized in cells even if nucleosides are damaged or altered. The presence of dG in all four mutation types of mirror-image nucleosides may be because dG is less sterically hindered and is better able to fill the gap (Fig. 3, *E*–*H*). The mutation process also occurs near the damaged site, suggesting that damage to a single nucleoside during repair not only results in the repair of a single site but also affects neighboring and surrounding bases. For this phenomenon, there is a resemblance to the observation made in transcriptional bypass experiments with N^2^-alkyl-2′-deoxyguanosine adducts (14).

When mirror-image nucleosides are present on the TS of an actively transcribed gene, mRNA exhibits significant base misincorporations, with adenosine or cytidine being inserted at that position. Due to that, we found more enriched entries for RNA polymerase II in the GO enrichment of the transcriptome and verified it by qPCR. We therefore speculate that L-dA strongly inhibits RNAPII (Fig. 5*A*), resulting in a lower abundance of full-length transcripts and moderate mutagenicity. Besides, L-dC has a weaker inhibitory effect on RNAPII shows a higher bypass efficiency, and exhibits mixed mutagenic properties during transcription (Fig. 5*C*). Among these types, dT is the most abundant mutagenic type (Fig. 5, *E*–*G*). This is presumably because of its unique chemical properties, which convert dTMP to UMP (RNA) during transcription. UMP is easily inserted into the transcribed mRNA by mistake due to the deletion of the methyl group (24). In addition to this, since we introduced base pairs that do not match the mirror-image nucleosides that are on the TS most mirror-image nucleosides are corrected to complementary base pairs of mismatched counterpoint bases. This phenomenon results in the highest replacement rates of the four mirror-image nucleosides on the TS being the *para*-complementary base pairs (L-dA→dG, L-dT→dG, L-dC→dA, and L-dG→dC), followed by the enantiomers of the L-configuration nucleosides. This experimental error caused by plasmid self-replication is excluded as the plasmid cannot replicate in HepG2 cells. Deletions of nucleoside sequences in mRNA were also observed. Indeed, one study found that lesion sites during DNA resynthesis may result in the observation of base misincorporation or deletion of full-length transcripts (25). Additionally, repair pathway, such as homologous recombination (26), mismatch repair (27), or nucleoside excision repair (28), may be involved, which all have the potential to result in the deletion of DNA fragments. L-dC on the TS is used as an example, and the position opposite to L-dC is dT. The highest mutation rate is L-dC→dA in all mutation results, which may be attributed to complementary base pair at opposite of mirror nucleotides. We speculated that mirror nucleotides could be excised *via* the nucleotide excision repair pathway or other mechanisms. And the gap was resynthesized using the complementary strand of the strand containing the mirror nucleotide as a template. However, this repair may occur before the mirror nucleotide starts transcription, and some unrepaired mirror nucleotides are repaired during transcription, resulting in L-dC→dC. Transcriptional mutagenesis are considered one of the major triggers for cancer and other human diseases, although the direct evidence, which links transcriptional mutagenesis to cancer, is currently lacking (29, 30, 31). However, studies have shown that mutation bypass of 8-oxo-deoxyguanine during transcription may activate carcinogenic pathways (32). Despite the relatively low mutation rate of mirror-image nucleosides during transcription compared to 8-oxo-deoxyguanine, mirror-image nucleosides still have the potential to activate certain pathogenic routes. This can result in detrimental drug side effects with prolonged usage.

**Supporting Figure S14:**
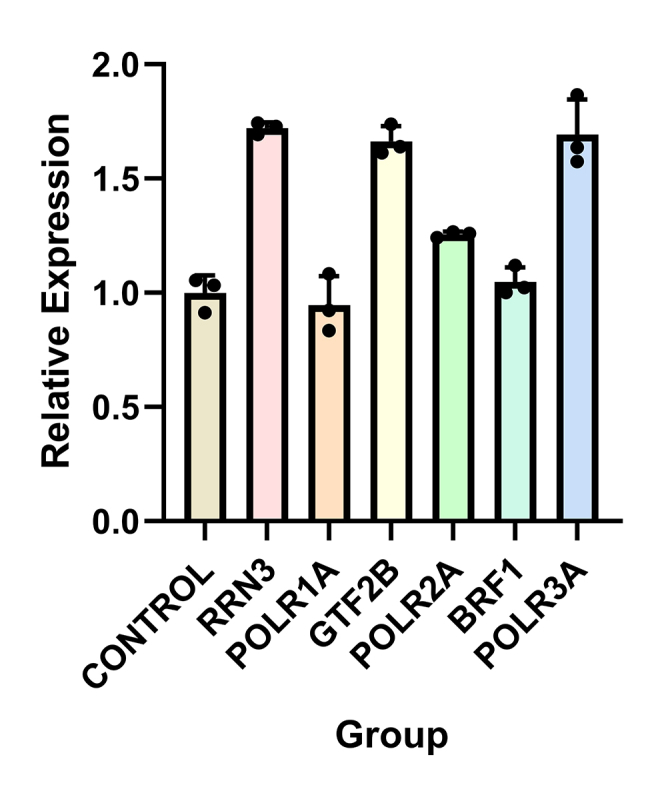


To study the above phenomenon, we transfected cells with plasmids containing L-dC and performed transcriptome analysis. The potential mechanism of mirror-image nucleoside repair *in vivo* was elucidated to provide a scientific basis for the study of antiviral drugs. The GO enrichment analysis revealed that the repair of mirror-image nucleosides is associated with RNA polymerase II proximal promoter sequence-specific DNA binding. Among the upregulated genes, JUN, BATF2, IFI16, and RELB were identified (Fig. S13*C*). JUN (33) is known to be involved in the positive regulation of transcription from RNA polymerase II promoter and cellular response to chemical stimulus. BATF2, RELB, and IFI16 are involved in the transcriptional regulation of RNA polymerase II. The results suggest a correlation between lesion repair during transcription of mirror-image nucleosides and RNA polymerase II. Subsequently, to ascertain any discernible alteration in RNA polymerase during the repair of mirror-image nucleosides, we conducted direct amplification of pertinent genes involved in the pathways of action for RNA polymerase I, II, and III using qPCR. These genes include RRN3, POLR1A, GTF2B, POLR2A, BRF1, and POLR3A. The results showed that the RNA polymerase-related gene expression levels increased slightly, but the changes were not significant (Fig. S14). We speculate that the repair of mirror-image nucleosides is a result of conformational changes in the polymerase during opening and closing that lead to changes in activity and thus affect the repair efficiency. The polymerase can bind to DNA in the open conformation, whereas in the closed conformation, it is unable to bind to DNA or react accordingly, and therefore the change in gene expression levels is not significant (13). We then performed KEGG pathway enrichment of upregulated genes and analyzed the JAK–STAT signaling pathway (34), which was enriched in both TM or RM experiments, and the key nodes of this signaling pathway were upregulated with related genes (SOCS3, JAK2, IL6, and STAT1) (Fig. S13*A*). The SOCS family of proteins is part of a classical negative feedback system that regulates cytokine signaling. SOCS3 (35) is involved in the negative regulation of cytokines signaling through the JAK–STAT pathway. JAK2 first binds to IL6 and immediately afterward signals to STAT proteins which activate the downstream pathway. SOCS is also a target gene of STAT (STAT promotes the transcriptional expression of SOCS) but SOCS in turn can exert a negative feedback regulation on the phosphorylation of STAT phosphorylation, thus forming a negative feedback chain of regulation. It was therefore hypothesized that when transfected with a plasmid containing mirror-image nucleosides into cells, it would lead to an inflammatory response that would activate the inflammatory signaling pathway of JAK-STAT stress. Indoleamine 2,3-dioxygenase 1 was found to be significantly upregulated in the RNA-Seq results, which is involved in peripheral immune tolerance (36) and helps maintain homeostasis *in vivo* by preventing uncontrolled and excessive immune responses. In conclusion, the integration of transcriptome sequencing results and qPCR results suggests that the repair of L-dC–containing plasmids is associated with immune regulation. It is hypothesized that when the mirror-image nucleoside enters the cell, it may cause a stressful inflammatory signaling pathway leading to an immune response, which removes a portion of the mirror-image nucleoside and causes a break in the DNA strand, immediately followed by inactivation of the JAK–STAT signaling pathway (37). The remaining mirror-image nucleoside-containing DNA strand that is not cleared is slowly repaired by TLS DNA polymerase or RNA polymerase II to produce a complete full-length transcript. Occasionally, a small number of mutations are generated during resynthesis due to some mismatches caused by poor correction.

Based on the aforementioned results, this study demonstrates that, in both the NTS and TS, L-dA acts as a formidable obstacle, exerting a potent inhibitory effect on replication or transcription, whereas L-dC exhibits the lowest fidelity among the four nucleosides and has a small steric hindrance. Moreover, mirror-image nucleosides elicit the induction of immune responses within cells, utilizing the JAK–STAT signaling pathway for immune modulation. These findings indicate that mirror-image nucleosides may exhibit dual effects: one blocks DNA or mRNA elongation, inhibiting gene replication and transcription, while the other induces mutations through mismatching, leading to drug side effects. Consequently, this study enhances our understanding of the cellular toxicity associated with nucleoside analog drugs.

## Material and methods

### Synthesis of ODN strands

Four 18-mer oligodeoxynucleotides (ODNs) containing L-dA, L-dT, L-dC, and L-dG were synthesized with a DNA synthesizer (K&A H-6). Deprotection and cleavage of the ODNs from the CPG support were performed overnight in concentrated ammonia. DNA purification was carried out using analytical and preparative HPLC (Agilent Technologies) with Agilent Technologies analytical columns (4.6 × 100 mm, 2.7 Micron). The applied buffer system consisted of 0.1 M triethylamine acetate aqueous solution and acetonitrile. The purity of the fractions was examined by analyzing HPLC. Purified ODNs were concentrated using a vacuum concentrator (Thermo Fisher Scientific), followed by deprotection and desalting, and validated by MALDI-TOF mass spectrometry. L-nucleoside phosphoramidite monomers for solid-phase DNA synthesis were purchased from Suzhou Biosyntech Co., Ltd and incorporated into DNA under standard conditions. Unmodified ODNs were purchased from Sangon Biotech (Sangon).

### Construction of a vector containing a single adduct

The pDsRed-monomer-N1 plasmid was obtained from MiaoLingBio. The pZAJ-5c-AGC construct was generated with the assistance of VectorBuilder Company. The obtained plasmids were sequenced, and protein expression and lifespan were validated in transfected cells. The human eukaryotic expression vector pZAJ-5c was constructed based on the pEGFP-C3 vector backbone (GenBank accession number: U57607) (10, 11, 12). The following methods were used to synthesize the plasmids used in the transcriptional bypass experiments. The plasmid DNA (120 μg) was incubated with 45 U of Nb.Bpu10I (from Fermentas) at 37 °C for 2 h to completely digest the plasmid DNA. Then, 45 U of Nb.Bpu10I was added and incubated at 37 °C for 4 to 6 h. The plasmid DNA was fully excised, which was converted into the nicked plasmid DNA. Afterward, the enzyme was heat-inactivated at 80 °C for 20 min. The restriction digestion mixture was extracted with an equal volume of phenol:chloroform: isoamyl alcohol solution (25:24:1) and gently shaken by hand for approximately 5 min. The mixture was centrifuged at 4 °C at 15,000*g* for 10 min. The aqueous layer was precipitated with 2.5 volumes of 100% (v/v) ethanol and 0.1 volume of 3 M sodium acetate. The mixture was incubated at −20 °C for at least 30 min and then centrifuged at 4 °C at 15,000*g* for 60 min. The DNA pellet was gently washed with 500 μl of 70% (v/v) ethanol and centrifuged at 4 °C at 15,000*g* for 5 min. The supernatant was removed, and the DNA pellet was dried and resuspended in 100 μl of sterile deionized water. Each microgram of nicked plasmid DNA was mixed with an approximately 900-fold molar excess of 18-mer complementary ODN (EXH-TS) (Table S1) in 1 × T4 DNA Ligase Buffer buffer. The reaction mixture was heated at 85 °C for 5 min and then gradually cooled to room temperature. The generated 18-mer duplex ODNs were removed using a Centricon 30 spin filter (Millipore), and the gapped plasmid was purified using four changes of Tris-EDTA buffer. The process was repeated once again. The gapped plasmid from the previous step was mixed with a 45-fold molar excess of phosphorylated ODNs containing the lesions (TS-A-L, TS-T-L, TS-C-L, and TS-G-L). The reaction mixture was heated to 85 °C for 10 min in T4 DNA ligase buffer and slowly cooled to 4 °C (0.02 °C/s). The reaction was transferred to ice, and T4 DNA ligase (Thermo Fisher Scientific) (1 μg with 10 U T4 DNA ligase) was added to the reaction buffer. The mixture was incubated at 10 °C for 30 s and then at 30 °C for 30 s, 800 cycles, followed by heat inactivation (65 °C, 15 min) (38). The larger volume was concentrated using a Centricon 100 spin filter (Millipore). The ligation mixture was loaded onto a 0.8% (w/v) agarose gel in the presence of GelRed and run in 1 × TAE buffer at 120 V for approximately 20 min. The band corresponding to the supercoiled plasmid DNA was excised under a short-wavelength (470 nm) blue light source using a surgical blade. The intact supercoiled plasmid DNA was recovered from the gel using the Qiagen QIAquick Gel Extraction Kit according to the manufacturer's instructions. The obtained control group (no damage) plasmid was sequenced to verify the integrity of the plasmid. The synthesis efficiency was verified by Sanger sequencing.

To synthesize the replicative bypass experiment plasmid, the initial experimental steps were identical to the aforementioned experiment. However, the initial nicking enzyme Nb.Bpu10I was replaced with Nt.Bpu10I, which nicked the NTS. Additionally, the complementary ODN was changed from EXT-TS to EXH-NTS (Table S1). Subsequently, the inserted ODNs containing mutations were 18-mer ODNs (NTS-A-L, NTS-T-L, NTS-C-L, and NTS-G-L), along with their corresponding control groups. After obtaining intact supercoiled plasmid DNA, each 1 μg of plasmid was incubated with 1 U Nb.Bpu10I (Fermenta) at 37 °C for 2 h. Then, 1 U of Nb.Bpu10I was added and the mixture was further incubated at 37° C for 4 to 6 h and then 80 °C for 20 min, to transform the supercoiled plasmid into nicked plasmid. DNA purification was performed using the aforementioned manual purification method. After purification, each microliter of nicked plasmids containing damage was mixed with an approximately 900-fold molar excess of 18-mer complementary ODNs (EXH-TS) in 1 × T4 DNA ligase buffer. The reaction mixture was heated at 85 °C for 5 min and then gradually cooled to room temperature. Centricon 100 spin filter (Millipore) was used to remove the generated 18-mer duplex ODNs, and the gap plasmid was purified using four changes of Tris-EDTA buffer. This step was repeated once again. The synthesis efficiency was also verified using Sanger sequencing.

### Cell transfection

Cell transfection experiments were performed using the constructed vectors described above. Cells were seeded in 12-well plates at a density of 5 × 10^5^ HepG2 cells per well (96-well plates at a density of 1 × 10^5^ HepG2 cells per well) in high-glucose Dulbecco's modified Eagle's medium (Invitrogen) supplemented with 10% fetal bovine serum. Then, recombinant plasmids containing EGFP and pDsRed-monomer-N1 plasmid were cotransinfected at the molar ratio of 1:1. Due to the absence of SV40T antigen in HepG2 cells and L02 cells, plasmids will not replicate inside the cells, eliminating interference caused by replication.

### Confocal microscope observation

After 18 h of transfection, the 96-well plate was washed 2 to 3 times with cold Dulbecco's Phosphate-Buffered Saline (DPBS), fresh DPBS was added, and then the cells were detected by laser confocal microscopy (Leica) with DsRed excitation wavelength of 557 nm, emission wavelength of 592 nm, and EGFP excitation wavelength of 488 nm and emission wavelength of 507 nm.

### Flow cytometry

After 18 h of transfection, the 12-well plate was washed 2 to 3 times with cold DPBS, and fresh DPBS was added. Then, add an equal amount of 1% formaldehyde is used to fix the cells in the 12-well plate for analysis using flow cytometry, employing BD Bioscience FACSAria III and FlowJo software (https://www.flowjo.cn/download.html). Before generating the distribution map of EGFP fluorescence (FL1-H), cells without transfection were cut off by selective gating based on DsRed expression, and the average EGFP signal of each cell was determined as the median of the distribution. EGFP and DsRed are detected in separate fluorescence channels in a flow cytometer. DsRed serves as a marker for gating transfected cells, enabling accurate quantification of EGFP expression (30). To quantify the relative abundance of EGFP mutants and measure transcriptional mutagenesis (TM), the signal was normalized to the median fluorescence of a reference construct encoding full-length EGFP recorded in the same experiment. To assess RM rates, the efficiency of repair of TS midgaps was calculated using a template encoding conventional EGFP, and this repair efficiency was expressed as the ratio of the number of EGFP-positive cells to the total number of transfected cells (9).

### RNA extraction and reverse transcription-quantitative PCR

Total cellular RNA was isolated using TRIzol reagent (Invitrogen) according to the manufacturer's instructions. cDNA synthesis for PCR amplification was generated using the RevertAid First Strand cDNA Synthesis Kit (Fermentas). The efficiency of reverse transcription was confirmed using control 1.1 kb mRNA. Oligo (dT) 18 primer and control RNA were provided by the kit manufacturer. The cDNA samples were diluted 5-fold and analyzed by quantitative real-time PCR. In parallel, samples without reverse transcription (“no-RT” control) were analyzed to detect potential contamination of plasmid DNA. Real-time PCR was performed using the Applied Biosystems QuantStudio 6 Flex and Power SYBR Green PCR Master Mix (Invitrogen). Specific primers for EGFP were 5′-GACCACTACCAGCAGAACAC-3′ and 5′-CTAGTACTTGTACAGCTCGTCC-3′. Primers for DsRed-Monomer were: 5′-CCTCCACCGAGAAGCTGTA-3′ and 5′-TCCACCACGGTGTAGTCCT-3′. PCR was performed for 40 cycles, followed by melting curve analysis to confirm the specificity of PCR products. Relative EGFP gene expression of damaged plasmids in each cell line was calculated based on the ratio of average EGFP cDNA levels between cells transfected with damaged and undamaged plasmids. These ratios were normalized to the level of DsRed-monomer cDNA in the respective samples, serving as an internal reference for transfection, mRNA preparation, and reverse transcription efficiency.

### High-throughput sequencing

Target sequencing was performed with the assistance of TinyGen company. First, the sample was prepared for library construction using specific primers. The library construction involved a two-step PCR amplification method. In the first step, the target fragments were amplified using specific primers, and the amplified fragments were then subjected to gel purification. Subsequently, the purified products were used as templates for a second round of PCR amplification, to add the Illumina platform-required adaptors, sequence primers, and barcodes to both ends of the target fragments. Afterward, Illumina high-throughput sequencing was performed. The obtained paired-end reads were first sorted based on barcodes to distinguish each sample. Then, the sequences underwent quality control and filtering. They were assembled based on overlap relationships, and the assembled sequences underwent another round of quality control and filtering to obtain optimized sequences. The optimized sequences were subjected to amplicon sequence variant (ASV) analysis and taxonomic classification. Based on the ASV analysis results, a table was generated that showed the distribution of ASVs for each sample. The total count of sequences that matched the plasmid sequences at both ends was used as the denominator, and the count of sequences with different mutation types in each sample was used as the divisor to calculate the mutation rate.

Cells were transfected with recombinant plasmids containing L-dC (A207P, S206A), with cells transfected with normal plasmids serving as the control group. Total cellular RNA was isolated using TRIZOL reagent (Invitrogen) following the manufacturer's instructions. The isolated RNA was then shipped to BGI for transcriptome sequencing and basic data analysis, preserved on dry ice. After quality control of the samples, the clean data were aligned with the reference genome, generating 1.15 to 1.19 Gb of data per sample. Gene expression quantification was conducted based on the alignment results. Differential analysis of cell gene expression was performed using DESeq (https://biosys.bgi.com) to identify differentially expressed genes. Functional annotation databases such as GO and KEGG were utilized to obtain significant enrichment of functional information and related pathway information based on the genes differentially expressed between samples or groups.

## Data availability

The raw data for ASVs have been deposited in the Sequence Read Archive under the accession number PRJNA1147155. The RNA-Seq raw data generated in this study have been deposited in the Gene Expression Omnibus under the accession number GSE274758. These datasets are publicly available and can be accessed through the respective repositories.

## Supplementary data

This article contains supporting information.

## Conflict of interest

The authors declare that they have no conflicts of interest with the contents of this article.

## Figures and Tables

**Supporting Figure S1:**
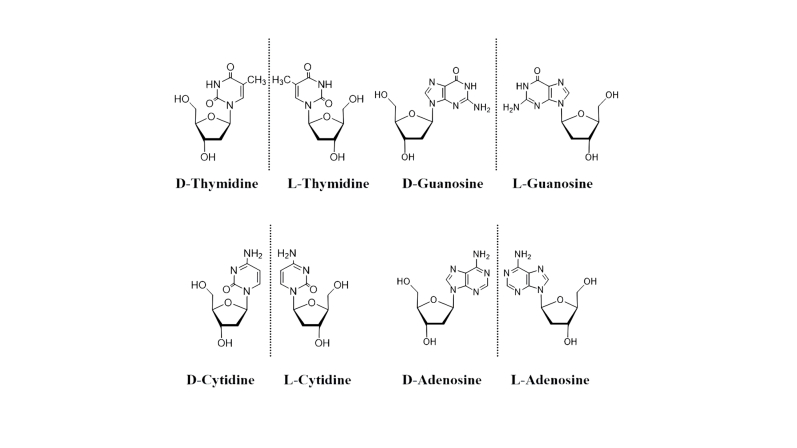


## References

[1] Torresi J., Locarnini S. (2000). Antiviral chemotherapy for the treatment of hepatitis B virus infections. Gastroenterology.

[2] Hsu Y.-C., Huang D.Q., Nguyen M.H. (2023). Global burden of hepatitis B virus: current status, missed opportunities and a call for action. Nat. Rev. Gastroenterol. Hepatol..

[3] Bryant M.L., Bridges E.G., Placidi L., Faraj A., Loi A.G., Pierra C. (2003). Antiviral β-L-nucleosides specific for hepatitis B virus infection. Front. Viral Hepat..

[4] Graves S.W., Johnson A.A., Johnson K.A. (1998). Expression, purification, and initial kinetic characterization of the large subunit of the human mitochondrial DNA polymerase. Biochemistry.

[5] Focher F., Maga G., Bendiscioli A., Capobianco M., Colonna F., Garbesi A. (1995). Stereospecificity of human DNA polymerases alpha, beta, gamma, delta and epsilon, HIV-reverse transcriptase, HSV-1 DNA polymerase, calf thymus terminal transferase and Escherichia coli DNA polymerase I in recognizing D- and L-thymidine 5'-triphosphate as substrate. Nucleic Acids Res..

[6] Jurovčík M., Holý A. (1976). Metabolism of pyrimidine L-nucleosides. Nucleic Acids Res..

[7] Jurovčík M., Holý A., Šorm F. (1971). The utilization of L-adenosine by mammalian tissues. FEBS Lett..

[8] Kan Y., Wu L., He Y. (2020). Mutation analysis of L-thymidine-induced replication products using a restriction enzyme–mediated assay. Curr. Protoc. Nucleic Acid Chem..

[9] Rodriguez-Alvarez M., Kim D., Khobta A. (2020). EGFP reporters for direct and sensitive detection of mutagenic bypass of DNA lesions. Biomolecules.

[10] Khobta A., Kitsera N., Speckmann B., Epe B. (2009). 8-Oxoguanine DNA glycosylase (Ogg1) causes a transcriptional inactivation of damaged DNA in the absence of functional Cockayne syndrome B (Csb) protein. DNA Repair.

[11] Lühnsdorf B., Kitsera N., Warken D., Lingg T., Epe B., Khobta A. (2012). Generation of reporter plasmids containing defined base modifications in the DNA strand of choice. Anal. Biochem..

[12] Allgayer J., Kitsera N., von der Lippen C., Epe B., Khobta A. (2013). Modulation of base excision repair of 8-oxoguanine by the nucleotide sequence. Nucleic Acids Res..

[13] Kan Y., Jin Z., Ke Y., Lin D., Yan L., Wu L. (2022). Replicative bypass studies of l-deoxyribonucleosides in Vitro and in E. coli cell. Scientific Rep..

[14] Tan Y., Guo S., Wu J., Du H., Li L., You C. (2021). DNA polymerase η promotes the transcriptional bypass of N2-Alkyl-2′-deoxyguanosine adducts in human cells. J. Am. Chem. Soc..

[15] Ji D., You C., Wang P., Wang Y. (2014). Effects of tet-induced oxidation products of 5-methylcytosine on DNA replication in mammalian cells. Chem. Res. Toxicol..

[16] Dimitri A., Burns J.A., Broyde S., Scicchitano D.A. (2008). Transcription elongation past O6-methylguanine by human RNA polymerase II and bacteriophage T7 RNA polymerase. Nucleic Acids Res..

[17] Nadkarni A., Burns J.A., Gandolfi A., Chowdhury M.A., Cartularo L., Berens C. (2016). Nucleotide excision repair and transcription-coupled DNA repair abrogate the impact of DNA damage on transcription. J. Biol. Chem..

[18] Jia N., Guo C., Nakazawa Y., van den Heuvel D., Luijsterburg M.S., Ogi T. (2021). Dealing with transcription-blocking DNA damage: repair mechanisms, RNA polymerase II processing and human disorders. DNA Repair.

[19] Sale J.E., Lehmann A.R., Woodgate R. (2012). Y-family DNA polymerases and their role in tolerance of cellular DNA damage. Nat. Rev. Mol. Cell Biol..

[20] Yang W. (2014). An overview of Y-family DNA polymerases and a case study of human DNA polymerase η. Biochemistry.

[21] Gaur V., Vyas R., Fowler J.D., Efthimiopoulos G., Feng J.Y., Suo Z. (2014). Structural and kinetic insights into binding and incorporation of L-nucleotide analogs by a Y-family DNA polymerase. Nucleic Acids Res..

[22] Jasti V.P., Spratt T.E., Basu A.K. (2011). Tobacco-specific nitrosamine-derived O2-alkylthymidines are potent mutagenic lesions in SOS-induced Escherichia coli. Chem. Res. Toxicol..

[23] Zhai Q.Q., Wang P.C., Cai Q., Wang Y.S. (2014). Syntheses and characterizations of the in vivo replicative bypass and mutagenic properties of the minor-groove O2-alkylthymidine lesions. Nucleic Acids Res..

[24] Bregenhorn S., Kallenberger L., Artola-Borán M., Peña-Diaz J., Jiricny J. (2016). Non-canonical uracil processing in DNA gives rise to double-strand breaks and deletions: relevance to class switch recombination. Nucleic Acids Res..

[25] Tornaletti S., Maeda L.S., Hanawalt P.C. (2006). Transcription arrest at an abasic site in the transcribed strand of template DNA. Chem. Res. Toxicol..

[26] Izhar L., Ziv O., Cohen I.S., Geacintov N.E., Livneh Z. (2013). Genomic assay reveals tolerance of DNA damage by both translesion DNA synthesis and homology-dependent repair in mammalian cells. Proc. Natl. Acad. Sci. U. S. A..

[27] Wu J.X., Gu L.Y., Wang H.X., Geacintov N.E., Li G.M. (1999). Mismatch repair processing of carcinogen-DNA adducts triggers apoptosis. Mol. Cell Biol..

[28] Yurchenko A.A., Padioleau I., Matkarimov B.T., Soulier J., Sarasin A., Nikolaev S. (2020). XPC deficiency increases risk of hematologic malignancies through mutator phenotype and characteristic mutational signature. Nat. Commun..

[29] Brégeon D., Doetsch P.W. (2011). Transcriptional mutagenesis: causes and involvement in tumour development. Nat. Rev. Cancer.

[30] Burns J.A., Dreij K., Cartularo L., Scicchitano D.A. (2010). O6-Methylguanine induces altered proteins at the level of transcription in human cells. Nucleic Acids Res..

[31] Dai D.P., Gan W., Hayakawa H., Zhu J.L., Zhang X.Q., Hu G.X. (2018). Transcriptional mutagenesis mediated by 8-oxoG induces translational errors in mammalian cells. Proc. Natl. Acad. Sci. U. S. A..

[32] Saxowsky T.T., Meadows K.L., Klungland A., Doetsch P.W. (2008). 8-Oxoguanine-mediated transcriptional mutagenesis causes Ras activation in mammalian cells. Proc. Natl. Acad. Sci. U. S. A..

[33] Potapova O., Basu S., Mercola D., Holbrook N.J. (2001). Protective role for c-Jun in the cellular response to DNA damage. J. Biol. Chem..

[34] Brooks A.J., Putoczki T. (2020). JAK-STAT signalling pathway in cancer. Cancers.

[35] Stärkel P. (2008). Genetic factors predicting response to interferon treatment for viral hepatitis C. Gut.

[36] van Baren N., Van den Eynde B.J. (2015). Tryptophan-degrading enzymes in tumoral immune resistance. Front. Immunol..

[37] Li T., Ge H., Yang Q., Wang J., Yin Q., Wang H. (2022). Oncogenic role of microRNA-19b-3p-mediated SOCS3 in glioma through activation of JAK-STAT pathway. Metabo. Brain Dis..

[38] You C.J., Wang Y.S. (2015). Quantitative measurement of transcriptional inhibition and mutagenesis induced by site-specifically incorporated DNA lesions in vitro and in vivo. Nat. Protoc..

