# Supplementary material for: Effects of mirror-image nucleosides on DNA replication and transcription in human cells
Source: J Biol Chem. 2024 Dec 26;301(2):108139. doi: 10.1016/j.jbc.2024.108139 (PMC11815684; doi:10.1016/j.jbc.2024.108139)
Supplement: Supporting information [file mmc1.docx]

**SUPPLEMENTAL MATERIAL**

**Effects of mirror-image nucleosides on DNA replication and transcription in** **human cells**

Zhaoyang Jin^1^, Yifei Wang^4^, Shuaishuai Cui^1^ and Yujian He^1,2,3*^, Li Wu^1,2*^

Includes:

Supplementary Table: 4

Supplementary Figures: 14

**Figure S1** The D-configuration versus the L-configuration nucleoside.

**Table S1** Single-stranded oligonucleotides used in this study. The red one is the L-type nucleotide insertion site.

| Name | Seq |
| --- | --- |
| TS-A-L | 5’-TCAGGGCGGACTA^[L]^GGTGC-3’ |
| TS-A-D | 5’-TCAGGGCGGACTAGGTGC-3’ |
| TS-G-L | 5’-TCAGGGG^[L]^GGACTGGGTGC-3’ |
| TS-G-D | 5’-TCAGGGGGGACTGGGTGC-3’ |
| TS-T-L | 5’-TCAGGGCGT^[L]^ACTGGGTGC-3’ |
| TS-T-D | 5’-TCAGGGCGTACTGGGTGC-3’ |
| TS-C-L | 5’-TCAGGGCGGC^[L]^CTGGGTGC-3’ |
| TS-C-D | 5’-TCAGGGCGGCCTGGGTGC-3’ |
| EXH-TS | 5’-GCACCCAGTCCGCCCTGA-3’ |
| NTS-A-L | 5’-TGAGCACCCAGTA^[L]^CGCCC-3’ |
| NTS-A-D | 5’-TGAGCACCCAGTACGCCC-3’ |
| NTS-G-L | 5’-TGAGCACCCAGG^[L]^CCGCCC-3’ |
| NTS-G-D | 5’-TGAGCACCCAGGCCGCCC-3’ |
| NTS-T-L | 5’-TGAGCACCT^[L]^AGTCCGCCC-3’ |
| NTS-T-D | 5’-TGAGCACCTAGTCCGCCC-3’ |
| NTS-C-L | 5’-TGAGCACCCAGTCCC^[L]^CCC-3’ |
| NTS-C-D | 5’-TGAGCACCCAGTCCCCCC-3’ |
| EXH-NTS | 5’-GGGCGGACTGGGTGCTCA-3’ |

**Figure** **S2** Oligonucleotide chains contain the synthesis of mirror image nucleosides. The left panel shows the liquid mass results and the right panel shows the gel electrophoresis results.

**Figure S3** (A) Plasmids (EG 1-7) extracted after amplification with *E. coli* were validated on agarose gels with control plasmids (CG) provided by the company. (B) pZAJ-5c was transformed into a nicked form using Nb. Bpu10I (TM).

**Figure S4** Results of sanger sequencing of control groups synthesized simultaneously with oligonucleotides containing the mirror nucleoside.

**Figure S5** (A) Amplified plasmid gel electrophoresis results. EG are the plasmid extracted from *E. coli.* CG is control plasmid provided by the company (B) The plasmid (pZAJ-5c) was transformed into a nicked form using Nt.Bpu10I. (C, D) Recombinant plasmids were obtained by ligating the synthesised oligonucleotides to the non-template strands (NTS) using T4 DNA ligase. (E, F) The closed-loop plasmid from the previous step was then recovered using gel recycling.

**Table S2** Nucleic acid strand deletion was found by target gene sequencing in RM and TM.

| L-dA-RM | CGTGCTGCTGCCCGACAACCACTACCTGAGC---------------------GCGATCACATGGTCCTGCTGGAG |
| --- | --- |
| L-dT-RM | CGTGCTGCTGCCCGACAACCACTACCTG-----------------------GCGCGATCACATGGTCCTGCTGGAG |
| L-dC-RM | CGTGCTGCTGCCC-------TGAGCAAAGACCCCAACGAGAAGCGCGATCACATGGTCCTGCTGGAG |
| L-dG-RM | CGTGCTGC-------CGCCCTGAGCAAAGACCCCAACGAGAAGCGCGATCACATGGTCCTGCTGGAG |
| L-dG-RM | CGTGCTGCTGCCCGACAACCACT--------------------------------GCGCGATCACATGGTCCTGCTGGAG |
| L-dT-TM | CGTGCTGCTGCCCGACAACCACTACCTGAGCACCCA--------------------------------GTCCTGCTGGAG |
| L-dT-TM | CGTGCTGCTGCCCGACAACCACTACCTGAGCACCCAGT------------------------TGGTCCTGCTGGAG |
| L-dC-TM | CGTGCTGCTGCCCGACAACCACTACCTGAGCACCCA------------------------------GGTCCTGCTGGAG |
| L-dG-TM | CGTGCTGCTGCCCGACAACCACTACCTGAGCACCCAGTCC-------------------ATGGTCCTGCTGGAG |

**Figure S6** Co-transfection of HepG2 cells with pZAJ and pDsRed-monomer-n1 plasmids was observed by confocal microscopy, and each plasmid was transfe-cted separately.

**Figure S7** Fluorescence intensity (relative to the original EGFP) measured in HepG2 cells transfected with plasmids containing specific L-nucleosides.

**Figure S8** Transcriptional bypass efficiencies of mirror image nucleotide in L02 cells.

**Table S3** Statistics of sequence data after filtering.

| **Sample Name** | **Clean Read** | **Clean Reads Ratio** |
| --- | --- | --- |
| **Control** | 23731034 | 99.93 |
| **RM-1** | 23733769 | 99.94 |
| **RM-2** | 23651083 | 99.60 |
| **TM-1** | 23730501 | 99.93 |
| **TM-2** | 23652410 | 99.60 |

**Figure S9** Sample correlation heat map. To depict the correlation of gene expression among samples, Pearson correlation coefficients for all gene expressions between each pair of samples were computed. These coefficients were visualized in a heat map, where higher values indicate greater similarity in gene expression levels across samples.

**Figure S10** Statistical table of the number of differential genes, A represents the results of the RM group versus the control group, and B represents the results of the TM group versus the control group.

**Figure S11** Plot of GO and KEGG enrichment results for up-regulated expressed genes after RM vs. control. A is GO enrichment and B is KEGG enrichment.

**Table S4** The 18 up-regulated DEGs

| Gene ID | Gene Symbol | Fold Change(RM) | Fold Change(TM) |
| --- | --- | --- | --- |
| [9021](https://biosys.bgi.com/#/report/gene-detail/F23A040004839_HOMgbldN/Animal/gene/nosts/9021) | SOCS3 | 1.1 | 1.22 |
| [8843](https://biosys.bgi.com/#/report/gene-detail/F23A040004839_HOMgbldN/Animal/gene/nosts/8843) | HCAR3 | 11.6 | 11.57 |
| [81856](https://biosys.bgi.com/#/report/gene-detail/F23A040004839_HOMgbldN/Animal/gene/nosts/81856) | ZNF611 | 0.92 | 1.24 |
| [6773](https://biosys.bgi.com/#/report/gene-detail/F23A040004839_HOMgbldN/Animal/gene/nosts/6773) | STAT2 | 2.57 | 2.51 |
| [6772](https://biosys.bgi.com/#/report/gene-detail/F23A040004839_HOMgbldN/Animal/gene/nosts/6772) | STAT1 | 2.65 | 2.59 |
| [6258](https://biosys.bgi.com/#/report/gene-detail/F23A040004839_HOMgbldN/Animal/gene/nosts/6258) | RXRG | 10.55 | 11.6 |
| [5971](https://biosys.bgi.com/#/report/gene-detail/F23A040004839_HOMgbldN/Animal/gene/nosts/5971) | RELB | 2.74 | 2.99 |
| [3725](https://biosys.bgi.com/#/report/gene-detail/F23A040004839_HOMgbldN/Animal/gene/nosts/3725) | JUN | 1.68 | 2.36 |
| [3717](https://biosys.bgi.com/#/report/gene-detail/F23A040004839_HOMgbldN/Animal/gene/nosts/3717) | JAK2 | 1.45 | 1.45 |
| [3659](https://biosys.bgi.com/#/report/gene-detail/F23A040004839_HOMgbldN/Animal/gene/nosts/3659) | IRF1 | 2.39 | 2.5 |
| [3569](https://biosys.bgi.com/#/report/gene-detail/F23A040004839_HOMgbldN/Animal/gene/nosts/3569) | IL6 | 5.93 | 6.49 |
| [3428](https://biosys.bgi.com/#/report/gene-detail/F23A040004839_HOMgbldN/Animal/gene/nosts/3428) | IFI16 | 2.74 | 2.76 |
| [10370](https://biosys.bgi.com/#/report/gene-detail/F23A040004839_HOMgbldN/Animal/gene/nosts/10370) | CITED2 | 1.75 | 1.74 |
| [11009](https://biosys.bgi.com/#/report/gene-detail/F23A040004839_HOMgbldN/Animal/gene/nosts/11009) | IL24 | 11.28 | 11.55 |
| [116071](https://biosys.bgi.com/#/report/gene-detail/F23A040004839_HOMgbldN/Animal/gene/nosts/116071) | BATF2 | 6.44 | 6.07 |
| [282618](https://biosys.bgi.com/#/report/gene-detail/F23A040004839_HOMgbldN/Animal/gene/nosts/282618) | IFNL1 | 10.89 | 10.89 |
| [338442](https://biosys.bgi.com/#/report/gene-detail/F23A040004839_HOMgbldN/Animal/gene/nosts/338442) | HCAR2 | 8.92 | 10.86 |
| [3620](https://biosys.bgi.com/#/report/gene-detail/F23A040004839_HOMgbldN/Animal/gene/nosts/3620) | IDO1 | 5.81 | 5.78 |

**Figure S12** Cluster heat map of gene expression levels

**Figure S13** Relative expression of differential genes in cells.

**Figure S14** Results of qpcr for genes associated with RNA polymerases I, II, and III in the TM
